# Complexation of Oligo- and Polynucleotides with Methoxyphenyl-Functionalized Imidazolium Surfactants

**DOI:** 10.3390/pharmaceutics14122685

**Published:** 2022-12-01

**Authors:** Darya A. Kuznetsova, Denis M. Kuznetsov, Leysan A. Vasileva, Syumbelya K. Amerhanova, Dilyara N. Valeeva, Diana V. Salakhieva, Viktoriia A. Nikolaeva, Irek R. Nizameev, Daut R. Islamov, Konstantin S. Usachev, Alexandra D. Voloshina, Lucia Ya. Zakharova

**Affiliations:** 1 Arbuzov Institute of Organic and Physical Chemistry, FRC Kazan Scientific Center, Russian Academy of Sciences, Arbuzov Str. 8, 420088 Kazan, Russia; 2 Institute of Innovation Management, Kazan National Research Technological University, Karl Marx Str. 68, 420015 Kazan, Russia; 3 Institute of Fundamental Medicine and Biology, Kazan (Volga Region) Federal University, Kremlyovskaya Str. 18, 420008 Kazan, Russia; 4 FRC Kazan Scientific Center of RAS, Russian Academy of Sciences, Lobachevsky Street 2/31, 420111 Kazan, Russia

**Keywords:** imidazolium surfactant, cationic surfactant, lipoplex, oligonucleotide, non-viral vectors, DNA

## Abstract

Interaction between cationic surfactants and nucleic acids attracts much attention due to the possibility of using such systems for gene delivery. Herein, the lipoplexes based on cationic surfactants with imidazolium head group bearing methoxyphenyl fragment (MPI-n, n = 10, 12, 14, 16) and nucleic acids (oligonucleotide and plasmid DNA) were explored. The complex formation was confirmed by dynamic/electrophoretic light scattering, transmission electron microscopy, fluorescence spectroscopy, circular dichroism, and gel electrophoresis. The nanosized lipoplex formation (of about 100–200 nm), contributed by electrostatic, hydrophobic interactions, and intercalation mechanism, has been shown. Significant effects of the hydrocarbon tail length of surfactant and the type of nucleic acid on their interaction was revealed. The cytotoxic effect and transfection ability of lipoplexes studied were determined using M-HeLa, A549 cancer cell lines, and normal Chang liver cells. A selective reduced cytotoxic effect of the complexes on M-HeLa cancer cells was established, as well as a high ability of the systems to be transfected into cancer cells. MPI-n/DNA complexes showed a pronounced transfection activity equal to the commercial preparation Lipofectamine 3000. Thus, it has been shown that MPI-n surfactants are effective agents for nucleic acid condensation and can be considered as potential non-viral vectors for gene delivery.

## 1. Introduction

Since the discovery of nucleic acids and elucidation of the pathogenesis mechanisms of some diseases, the replacement of defective genes with healthy ones has been considered as a new direction in science, which is called gene therapy [1,2]. Gene therapy can be considered as a tool for the transfer of nucleic acids such as oligonucleotides, pDNA, siRNA, or mRNA to treat numerous diseases [3,4,5,6]. The simplest form of treatment within gene therapy is to use “naked” DNA; however, there are a number of problems (large DNA size, enzymatic degradation by nucleases, clearance by the reticuloendothelial system) that can only be solved using gene carriers [7,8]. There are two types of vectors in gene therapy: viral and non-viral. It is known that viral vectors (adenoviruses, retroviruses, adeno-associated viruses, and herpes simplex virus) have a high ability of DNA transfection into the cell [9]. However, problems associated with viral vectors, including toxicity, immunogenicity, and limitations in large-scale use, have stimulated the search for new potential vectors for introducing DNA into target tissues [10]. Non-viral gene delivery is of great interest due to its excellent biosafety profile. High efficiency, transfection safety, and marked specificity contributed to the increase in the number of non-viral vectors in clinical trials [2]. Non-viral strategies can be divided into physical and chemical techniques [1]. Physical methods use physical force to create temporary pores in cell membranes, which allow DNA to enter cells via diffusion. Chemical methods use natural or synthetic compounds as carriers to deliver genetic material into the cell [1]. Numerous chemical compounds have been synthesized as potential non-viral vectors. The most traditional non-viral vectors are based on cationic lipids [11,12,13], but there are also reports on the use of cationic surfactants [14,15], peptides [16], ionic liquids [17], polymers [18,19], dendrimers [20], lipid nanoparticles [21], and poly(β-amino esters) [22]. In the last decade, cationic surfactants have been the object of numerous studies as potential gene carriers [23,24,25,26], since they are able to compact DNA molecules into small aggregates and protect them from degradation by nucleases [27]. As a result, they penetrate through cell membranes, releasing the genetic material [20]. In addition, studies conducted with various types of surfactants (anionic, nonionic, and cationic) have shown that cationic amphiphiles are the most effective agents for DNA condensation, causing a morphological transition of nucleic acid from an elongated form to a globular one [28,29,30,31]. Structural characteristics of surfactants strongly determine the nature of the interaction between amphiphiles and DNA. The interaction degree is affected by several parameters: (1) the charge on the surfactant head group [4,32]; (2) hydrophobic tail length and the presence of side chains in the surfactant structure [33,34]; (3) the presence of functional groups at the end of the hydrophobic tail [35].

**Figure 1:**
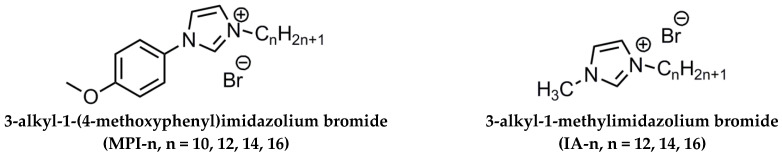
Homologous series of MPI-n (n = 10, 12, 14, 16) and IA-n (n = 12, 14, 16).

The high publication activity in this area indicates that the development of surfactant-based gene carriers remains an ongoing task. This is mainly due to the low transfection ability of synthetic amphiphiles compared with viral vectors. Therefore, the search for new carriers of nucleic acids, as well as the assessment of the role of various factors in the complex formation, is important area of research. Numerous researchers have demonstrated that cationic surfactants may be successfully used as drug and gene nanocarriers [36,37,38,39]. Aside from their high practical significance, these studies involving homologous series of cationic surfactants with different head groups provide important fundamental data on the structure–activity correlation. In the present work, new imidazolium surfactants with methoxyphenyl fragment and various hydrocarbon tail lengths have been explored as carriers for genetic material delivery (Figure 1). Oligonucleotide (ONu) and plasmid DNA pK18 were used as nucleic acids.

Previously, mainly ammonium cationic surfactants were used as non-viral vectors. Notably, few publications are devoted to the complexation of nucleic acids with imidazolium amphiphiles. Monomeric and gemini non-functionalized imidazolium surfactants are known to be able to effectively interact with oligonucleotides and DNA [40,41,42]. Strong complexation between the components protects DNA from enzymatic degradation [43]. The binding of imidazolium surfactants to nucleotide units occurs via several mechanisms, such as electrostatic binding, hydrophobic interactions, and intercalation π–π interaction. Moreover, the last two mechanisms play a dominant role in the complexation process. In those cases, the electrostatic binding is so weak that, even with a large excess of surfactants, no recharging occurs in the systems [40,41,42]. A similar trend is observed for hydroxyethylated imidazolium surfactants [44]. The functionalization of amphiphiles by the carbamate fragment changes the situation, and the electrostatic binding of the components begins to predominate [45].

Based on these considerations, it can be suggested that the inclusion of methoxyphenyl fragment into the imidazolium surfactant (Figure 1) can significantly affect the complexation of amphiphile/nucleic acid. Firstly, the appearance of an additional hydrophobic fragment in the surfactant structure can enhance binding in surfactant/oligonucleotide or surfactant/DNA binary systems. It was shown that amphiphiles with higher hydrophobicity (and lower critical micelle concentration (CMC)) are most effective at compacting DNA from an elongated structure to a globular one [34]. Secondly, the presence of oxygen in the methoxyphenyl fragment can provide additional hydrogen bonding of surfactants with nucleic acids.

## 2. Materials and Methods

### 2.1. Reagents and Sample Preparation

#### 2.1.1. Reagents and Probes

The chemicals were purchased from Sigma-Aldrich and Syntol and were used without prior purification. Ethidium bromide (EB) (Sigma-Aldrich, St. Louis, MI, USA, 95%) was used as a fluorescent probe. The following reagents were chosen as genetic material: commercial oligonucleotide M13F (Syntol, Moscow, Russia, 95–99%), which has the gta-aaa-cga-cgg-cca-gtg sequence and molecular weight of 5558 g/mol, plasmid DNA pK18 (2661 bp), which was isolated from bacterial cells using Plasmid Maxi Kit (Qiagen, Redwood, CA, USA) [46], plasmid DNA (pDNA) pEGFP-N2 (4737 bp) (Sigma-Aldrich, St. Louis, MI, USA). For experiments on a fluorescent microscope, oligonucleotides modified (Syntol, Moscow, Russia, 95–99%) with fluorescent labels of carboxy-X-rhodamine ((ROX)gt-aaa-acg-acg-gcc-agt-g, molecular weight is 6235 g/mol) and carboxyfluorescein ((FAM)gt-aaa-acg-acg-gcc-agt-g, molecular weight is 6095 g/mol) were used. Agarose for molecular biology was purchased from Sigma-Aldrich (Sigma-Aldrich, St. Louis, MI, USA).

The homologous series of MPI-n (n = 10, 12, 14, 16) was obtained via the reaction of 1-(4-methoxyphenyl)imidazole with the corresponding alkyl bromide [47,48]. Briefly: the mixture of 1-(4-methoxyphenyl)-1H-imidazole (1 mol) and corresponding alkyl bromide (1.1 mol) was stirred in dry acetonitrile under heating. After that, the solvent was evaporated in a vacuum. The pure product was obtained after adding a solvent mixture of hexane/ethyl acetate (4:1) for MPI-10; diethyl ether/ethyl acetate (2:1) for MPI-12; diethyl ether for MPI-14 and MPI-16. The precipitate was filtered, washed with diethyl ether, and dried in a vacuum.

#### 2.1.2. Solution Preparation

An aqueous solution of tris-HCl buffer (Sigma-Aldrich, St. Louis, MI, USA, 99%) with a concentration of 4 mM and pH 8.0 was used as a solvent for preparation of the solutions of ONu and DNA. Millipore Milli-Q deionized water (18.2 MΩ cm resistivity) was used to prepare surfactant solutions.

### 2.2. Methods

#### 2.2.1. Dynamic and Electrophoretic Light Scattering

The determination of hydrodynamic diameter (D_H_ = 2R_H_, nm) and zeta potential of ONu and DNA molecules, as well as their complexes with surfactants, was carried out on a ZetaSizer Nano particle analyzer (Malvern Instruments Ltd., Worcestershire, UK) [49,50,51]. The hydrodynamic radius of the particles (R_H_) was calculated based on the Stokes–Einstein Equation (1) [52,53,54]:

D = kT/(6πηR_H_) (1)

where k is the Boltzmann’s constant, T is the absolute temperature, η is the solvent’s viscosity, and R_H_ is the hydrodynamic radius.

The electrophoretic mobility of the samples was converted to zeta potential using the Smoluchowski Equation (2):

ζ = μη/ε(2)

where ζ is the zeta potential, η is the dynamic viscosity of the solution, μ is the mobility of the particle, and ε is the dielectric constant.

The experiment was carried out at an initial concentration of ONu equal to 500 µM and DNA equal to 10 µM and at sequential titration of these systems with surfactant solutions to obtain a certain molar ratio of N/P (nitrogen/phosphorus − *r*).

#### 2.2.2. Gel Electrophoresis

To obtain complexes, pDNA at a final concentration of 10 μg/mL was gently mixed with MPI-12 (20–650 μg/mL) or MPI-16 (10–325 μg/mL) in phosphate-buffered saline (PBS). The mixture was incubated at ambient temperature for 20 min. Electrophoretic analysis of pDNA–surfactant complexes was performed in 1% agarose gel in Tris-acetate–EDTA buffer using electrophoresis apparatus (Bio-Rad Laboratories, Hercules, CA, USA). Electrophoresis conditions were detailed previously [55]. Briefly, pDNA (200 ng per well) was separated at a voltage of 8 V/cm for 60 min and then stained with ethidium bromide (0.5 μg/mL). The gels were visualized using a ChemiDoc XRS Plus gel documentation system (Bio-Rad Laboratories, Hercules, CA, USA). O’Gene Ruler DNA Ladder Mix (100–10,000 bp) (Fermentas, Waltham, MA, USA) was used.

#### 2.2.3. Fluorescence Spectroscopy

The fluorescence intensity of EB/ONu and EB/DNA complexes was studied on a Hitachi F-7100 (Hitachi High-Tech Corporation, Minato, Tokyo, Japan) spectrofluorimeter. The excitation wavelength for EB was set at 480 nm; the fluorescence spectrum was recorded in the range from 500 to 700 nm. The solutions with a volume of 700 µL (the EB concentration is 0.5 µM; ONu or DNA concentration is 10 µM) were prepared directly in cuvette and equilibrated at 25 °C for 20 min. Next, an aliquot of the surfactant stock solution was gradually added to the EB/ONu or EB/DNA complexes, and the fluorescence spectrum was recorded. The titration was terminated when pronounced fluorescence quenching was achieved.

Equation (3) [44] was used to estimate the binding degree of ONu or DNA (*β*) to surfactant:

(3) $$\beta =\frac{\left(I_{b}-I_{obs}\right)}{\left(I_{b}-I_{f}\right)}$$

where *I_f_* and *I_b_*—fluorescence intensity of free EB and EB bound with ONu or pK18; *I_obs_*—fluorescence intensity at certain MPI-n/ONu or MPI-n/pK18 molar ratio.

#### 2.2.4. Circular Dichroism Experiments

Circular dichroism (CD) experiments were carried out on a Jasco J-1500 spectrometer (Jasco, Tokyo, Japan) at 25 °C. CD spectra were recorded in the wavelength range from 220 to 320 nm in 10 mM sodium-phosphate buffer, pH 7.4. The scanning speed was 50 nm/min; three scans were averaged. The optical path length of the used quartz cuvette was 1 cm. The initial concentration of the oligonucleotide was 50 μM per nucleotide unit. The stock oligonucleotide solution was titrated with concentrated surfactant solutions (5 mM) and the CD spectra were recorded. Before each measurement, the complexes were incubated for 5 min. Dilution factors were taken into account to calculate the final charge factors. The resulting spectra were expressed in terms of molar ellipticity.

#### 2.2.5. Transmission Electron Microscopy

The MPI-n/ONu complexes were visualized via transmission electron microscopy (TEM) on a Hitachi HT7700 instrument (Hitachi High-Technologies Corporation, Tokyo, Japan). The photographs were taken at an accelerating voltage of 100 kV. Samples were dispersed on 300 mesh copper grid with continuous carbon–formvar support films [56,57].

#### 2.2.6. Cytotoxicity Assay

The cytotoxic effect of the MPI-n/ONu and MPI-n/pK18 complexes was studied using MTT Assay. The cytotoxic effect on cells was determined using the colorimetric method of cell proliferation—the MTT test. NADP-H-dependent cellular oxidoreductase enzymes can, under certain conditions, reflect the number of viable cells. These enzymes are able to reduce the tetrazolium dye (MTT)—3-(4,5-dimethylthiazol-2-yl)-2,5-diphenyl-tetrazolium bromide to insoluble blue–violet formazan, which crystallizes inside the cell. The amount of formazan formed is proportional to the number of cells with active metabolism. Cells were seeded on a 96-well Nunc plate at a concentration of 5 × 10^3^ cells per well in a volume of 100 μL of medium and cultured in a CO_2_ incubator at 37 °C until a monolayer was formed. Then, the nutrient medium was removed and 100 μL of solutions of the test drug in the given dilutions was added to the wells, which were prepared directly in the nutrient medium with the addition of 5% DMSO to improve solubility. After 24 h of incubation of the cells with the tested compounds, the nutrient medium was removed from the plates and 100 µL of the nutrient medium without serum with MTT at a concentration of 0.5 mg/mL was added and incubated for 4 h at 37 °C. Formazan crystals were added 100 μL of DMSO to each well. Optical density was recorded at 540 nm on an Invitrologic microplate reader (Novosibirsk, Russia). The experiments for all compounds were repeated three times.

Lung adenocarcinoma (A549), cervical cancer (M-HeLa) were obtained from the collection of type cultures of the Institute of Cytology (The Russian Academy of Sciences, Saint Petersburg, Russia) [58,59]. Human normal liver cell lines (Chang liver) was obtained from the collection the Research Institute of Virology of the Russian Academy of Medical Sciences (Moscow, Russia).

#### 2.2.7. Flow Cytometry Assay

Cellular uptake of the MPI-n/ONu complexes was analyzed on a flow cytometer (Guava easy Cyte 8HT, Luminex Corporation, Austin, TX, USA). Fluorescently labeled oligonucleotides were used for the experiments. Briefly: cells at a concentration of 1 × 10^5^ cells/well were plated in 24-well plates (Eppendorf, Hamburg, Germany). Then, the cells were incubated for 24 h and the MPI-n/ONu complexes (N/P ratio is 1:1) of various concentrations were added. Further incubation was carried out for 24 h in a CO_2_ incubator and analysis of cellular uptake. Untreated cells were used as a negative control [58,60].

#### 2.2.8. Fluorescence Microscopy

Normal and cancer cells were seeded in 6-well plates with coverslips on the bottom (cell count 1 × 10^5^ cells/well in a final volume of 2 mL). The cells were incubated for 24 h and MPI-n/ONu complexes were added to the wells (N/P ratio is 1:1). The cells were then incubated again for 24 h in a CO_2_ incubator. After treatment, the cells were fixed and stained with DAPI (blue). Imaging was performed using a Nikon Eclipse Ci-S (Nikon, Nanjing, China) fluorescent microscope [58].

#### 2.2.9. DNA Transfection

A549 and M-HeLa cells were transfected with pDNA encoding green fluorescent protein (pEGFP-N2). The cells were seeded into 24-well plate at a density of 50,000 cells per well and cultured for 24 h in full α-MEM medium. Then, the culture medium was replaced with the fresh one with 0 or 10% fetal bovine serum (FBS), and preformed MPI-n/pDNA complexes were added to the cells at an amount of pDNA of 500 ng per well. Lipofectamine 3000 transfection reagent was used according to the manufacturer’s protocol (Invitrogen). The cells were incubated with the complexes for 4 h in a CO_2_ incubator, followed by replacement of the transfection medium with full α-MEM and further cell culturing for 24 h. The treated cells were collected by trypsinization, and the transfection efficiency was analyzed by measuring green fluorescence in the cells on a Guava EasyCyte8HT (Luminex Corporation, Austin, TX, USA) flow cytometer.

#### 2.2.10. Hemagglutination Assay

In the experiment, human erythrocyte mass was used (Group IV). The ability of MPI-n/ONu complexes to induce hemagglutination was tested using a 2% suspension of erythrocytes in 96-well U-plates. Various N/P molar ratios for the MPI-12/ONu complexes (r = 0.6; r = 1; r = 2) were used for the experiments. Previously, erythrocytes were washed twice with 0.9% saline and centrifuged at 2500× *g* for 10 min at 4 °C. After each cycle, the supernatant was carefully removed. The erythrocytes were then resuspended in 0.9% saline to a concentration of 2%.

For the study of hemagglutination, a series of MPI-12/ONu solutions with double dilution was prepared. Then, 100 µL of the complex solution was mixed with 100 µL of a 2% erythrocyte solution and placed in a well. The experiment was carried out in two parallel wells. After mixing the components, incubation was carried out for 1 h at 37 °C. Hemagglutination was observed with the naked eye [61] and assessed using a Nikon Eclipse Ci-S fluorescent microscope (Nikon, Nanjing, China). A suspension of erythrocytes in 0.9% NaCl and a mixture of type A (II) and B (III) erythrocytes were used as negative and positive control of agglutination, respectively [51].

#### 2.2.11. X-ray Crystallography

Data for single-crystal MPI-12 were collected on a Rigaku XtaLab Synergy S instrument with a HyPix detector and a PhotonJet microfocus X-ray tube using Cu Kα (1.54184 Å) radiation at low temperature. Images were indexed and integrated using the CrysAlisPro data reduction package (Woodstock Rd, Kidlington, UK). Data were corrected for systematic errors and absorption using the ABSPACK module: numerical absorption correction based on Gaussian integration over a multifaceted crystal model and empirical absorption correction based on spherical harmonics, according to the point group symmetry using equivalent reflections. The GRAL module was used for analysis of systematic absences and space group determination. The structure was solved by direct methods using SHELXT (Boston, MA, USA) [62] and refined by the full-matrix least-squares on F2 using a SHELXL (Boston, MA, USA) [63]. Non-hydrogen atoms were refined anisotropically. The hydrogen atoms were inserted at the calculated positions and refined as riding atoms. The figures were generated using a Mercury 4.1 [64] program (Karlsruhe, Germany). Crystals were obtained by slow evaporation method.

## 3. Results and Discussion

### 3.1. DLS Measuring for Surfactant/ONu and Surfactant/DNA Complexes

DNA and other nucleic acids are hydrophilic, negatively charged macromolecules. As a rule, in aqueous solutions, DNA is in the state of an elongated coil. However, in case of obtaining lipoplexes capable of penetrating inside the cells, it is necessary to transform the DNA into a compact form [34]. Compaction of DNA chains can be induced by adding complexing agents, such as cationic surfactants [28,65]. Therefore, in order to simulate the interaction of cationic amphiphiles with DNA, the complex formation of an oligonucleotide consisting of 18 nucleotide units with imidazolium surfactants containing methoxyphenyl fragment was studied. Particle size is the most important factor in genetic engineering, as it affects elimination, penetration into tumor cells, and retention in various organs and tissues due to variable threshold sizes in different locations [66]. In addition, it is important that the particles are not taken up by the mononuclear phagocytic system. It has been shown that optimal circulation time, which correlates with high uptake by tumor cells, is achieved for particles with a hydrodynamic diameter of 70–200 nm [67]. Additionally, some studies have shown that the particle size for gene delivery should not exceed 150 nm [68].

**Figure 2:**
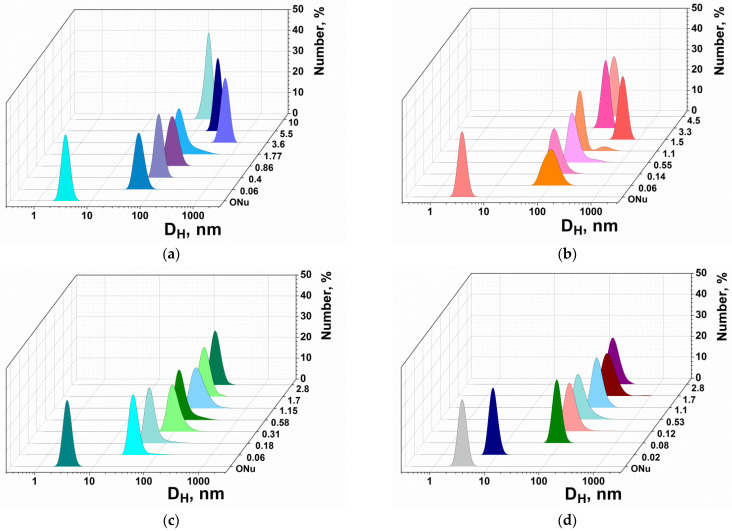
Number-averaged size distribution of the MPI-n/ONu complexes at different *r*: (**a**) MPI-10/ONu, (**b**) MPI-12/ONu, (**c**) MPI-14/ONu, (**d**) MPI-16/ONu; 25 °C. The colors shown represent different *r* values.

A dynamic light scattering method was used to determine the sizes of the MPI-n/ONu complexes. The size of lipoplexes (surfactant/ONu complexes) was estimated by varying the molar ratio of the components N/P (*r*). The results are shown in Figure 2 and Table S1. As shown, an individual oligonucleotide has a hydrodynamic diameter (D_H_) of about 2–6 nm. The addition of surfactants, even at low concentrations, initiated the formation of larger mixed complexes (about 50–200 nm). It should be noted that, during the formation of the MPI-n/ONu complexes, the variation in the molar ratio slightly affects the aggregate sizes. However, for the MPI-10/ONu and MPI-12/ONu systems, large particles with D_H_ of about 500–600 nm were observed near the isoelectric point, which is probably due to particle agglomeration. A further increase in the surfactant concentration led to compaction of the aggregates up to 100–150 nm in diameter. MPI-n/ONu complexes had an optimal size in almost the entire range of concentrations studied.

**Figure 3:**
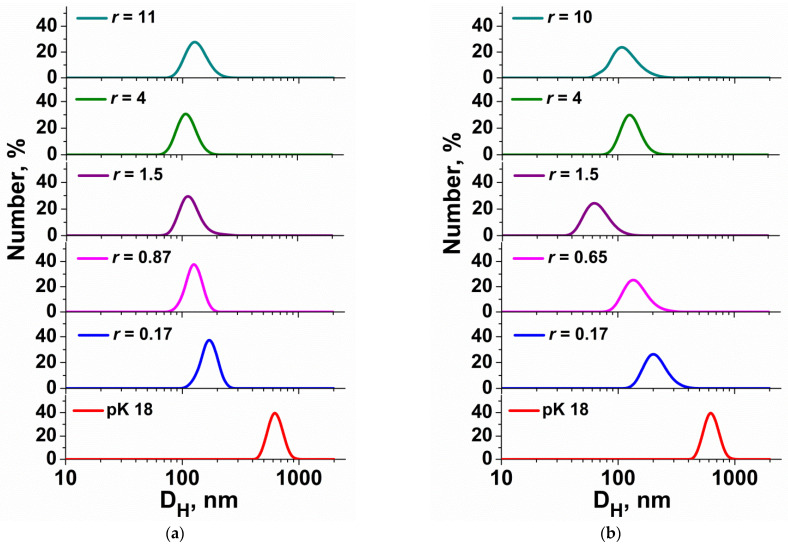
Number-averaged size distribution of the MPI-10/pK18 (**a**) and MPI-12/pK18 (**b**) complexes at different molar ratios; 25 °C.

Further, a transition was made from an oligonucleotide consisting of 18 nucleotide units to plasmid DNA pK18 (2661 base pairs). It was interesting to check whether the patterns of complexation established for surfactant/ONu systems correlate with surfactant/DNA systems. Figure 3 and Figure S1 show the dimensions of DNA and surfactant/DNA complexes obtained via dynamic light scattering. It can be seen that the size of an individual DNA pK18 macromolecule is at the level of 500–800 nm. The addition of cationic surfactants in minimal concentrations initiates the formation of mixed complexes of a more compact size (approximately 80–200 nm). It should be noted that the size of complexes of ≤200 nm meets the criteria for nanocontainers for the biotransport of therapeutic agents and probes [67]. In the case of an ONu, the size of the complexes increased with the addition of surfactant (up to 100–200 nm) due to the formation of vesicle-like aggregates. In the case of pK18 plasmid, the addition of surfactant resulted in the compaction of the macromolecule, and a further increase in the amphiphile concentration had almost no effect on the size of lipoplexes. This is probably due to the fact that surfactant kept the plasmid in a relatively compact package due to the neutralization of phosphate anions.

### 3.2. Zeta Potential for Surfactant/ONu and Surfactant/DNA Complexes

**Figure 4:**
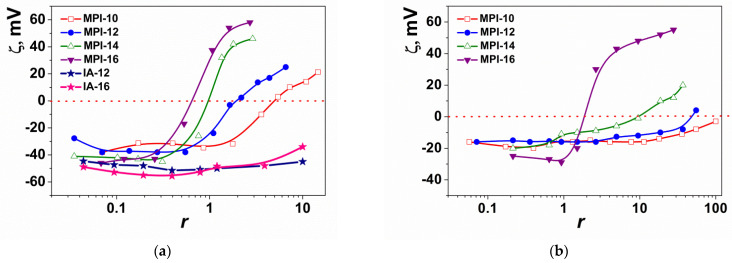
Zeta potential of the surfactant/ONu (**a**) and surfactant/pK18 (**b**) complexes as function of *r* value; 25 °C.

Further studies were conducted to evaluate the zeta potential of the systems as a function of *r*. The zeta potential of MPI-n/pK18 and MPI-n/ONu lipoplexes was measured by electrophoretic light scattering. An individual ONu has a negative charge (approximately −40 mV). The addition of cationic surfactants (MPI-n) leads to electrostatic binding of the components, as evidenced by the increase in the zeta potential of the complexes and its transition from the negative to the positive range (Figure 4a). The length of the alkyl chain significantly affects the value of *r* corresponding to zero potential. The longer the hydrocarbon tail of the surfactant and the lower its critical micelle concentration (CMC), the lower the concentration when recharging occurs in the system. It is interesting to compare these results with non-functionalized imidazolium surfactants (IA-n, Figure 1) and imidazolium surfactants with a hydroxyethyl moiety (IA-n(OH)) [40,41,44]. For the amphiphiles mentioned, recharging in surfactants/ONu systems was not achieved even at a large excess of surfactants, regardless of the hydrocarbon tail length. Figure 4a exemplifies the zeta potential dependence on *r* for the IA-12/ONu and IA-16/ONu systems. It can be seen that for non-functionalized imidazolium surfactants (IA-n), the hydrocarbon tail length does not affect the charge changes in the surfactant/ONu system.

As in the case of the ONu (Figure 4a), during the complex formation of amphiphiles with the plasmid pK18, compensation of the negative DNA charge was observed (Figure 4b). However, the recharging of the complexes did not occur in every instance. For the MPI-10/DNA system, even with a large excess of surfactants, it was not possible to reach the isoelectric point. Additionally, for other homologues, the charge exchange in surfactant/DNA systems occurred at higher *r* than in the case of surfactant/ONu systems. Recharging in the MPI-12/DNA system was achieved at *r* = 52 (at *r* = 1.8 for MPI-12/ONu), in the MPI-14/DNA system at *r* = 9.5 (at *r* = 1 for MPI-14/ONu), in the MPI-16/DNA system at *r* = 1.8 (at *r* = 0.65 for MPI-16/ONu). Such a charge inversion is caused by compaction and condensation of DNA, which is associated with the binding of amphiphile molecules with nucleic acid and is due to electrostatic interactions [33,34]. It should be taken into account that, despite the higher N/P charge exchange ratio for complexes with pK18, the absolute concentrations of amphiphiles were much lower than in the case of ONu. This allows us to conclude that the surfactant binding capacity in relation to the pK18 plasmid is high and MPI-16 can be considered as effective complexing agents.

### 3.3. Gel Electrophoresis

**Figure 5:**
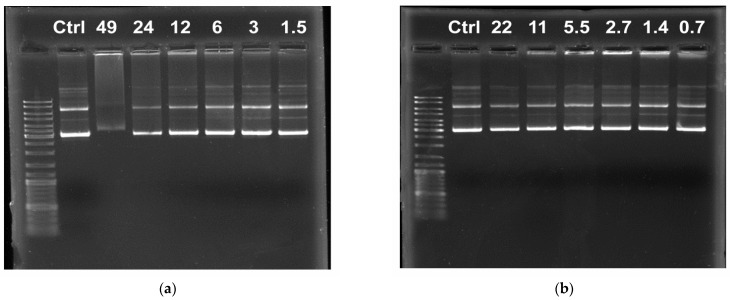
Electrophoretic mobility of pDNA in complex with MPI-12 (**a**) and MPI-16 (**b**). Ctrl shows pure pDNA; N/P ratios are indicated above corresponding wells. DNA ladder from 100 to 10,000 bp was used.

To test the ability of surfactants to bind to DNA, electrophoretic analysis was also performed with the use of pDNA (Figure 5). The experiment was carried out for amphiphiles with dodecyl (MPI-12) and hexadecyl (MPI-16) tail. According to electrophoretic data, MPI-12 showed pDNA-binding activity at a concentration of 650 µg/mL (mass stoichiometry of complexes = 65, N/P = 49, Figure 5a). Whereas MPI-16 did not retard pDNA at upper concentration of 325 µg/mL (mass stoichiometry of complexes = 32, N/P = 22, Figure 5b), which was close to its maximum soluble concentration. It should be noted, that when the mass stoichiometry of the MPI-16/pDNA complex was 32 (N/P = 22), turbidity was observed, which probably indicated a certain degree of aggregation of the complex. Interaction of MPI-16 with pDNA is testified by the appearance of pDNA in the wells, as if part of the pDNA lost electrophoretic mobility due to the formation of a complex with MPI-16.

### 3.4. Single-Crystal X-ray Investigations of the Structure of the MPI-12

**Figure 6:**
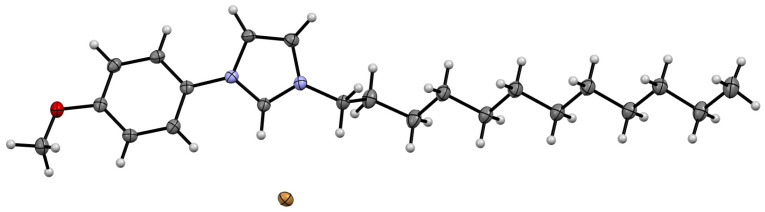
ORTEP representation of MPI-12 showing 50% probability thermal ellipsoids. C atoms—grey, Br atoms—brown, O atoms—red, N atoms—blue.

**Figure 7:**
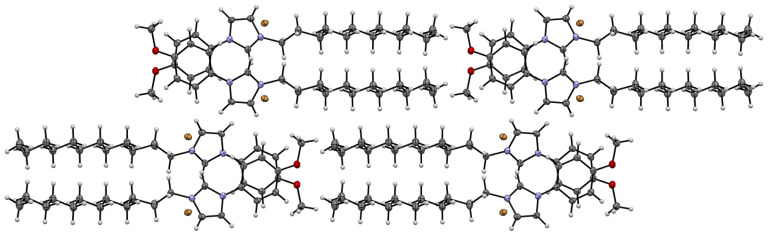
A partial view along the *c* axis of the crystal packing of MPI-12.

In order to elucidate the reason for the different behavior of surfactants (MPI-n and IA-n) in complexes with ONu, the X-ray single-crystal diffraction method was used. In order to grow a crystal, the MPI-12 system was chosen as an example. The crystals were obtained by vapor diffusion using a binary solvent system: ethyl acetate/diethyl ether. Single-crystal XRD analysis of the MPI-12 compound showed that this compound is a salt, in which [C12MPI]^+^ acts as a cation, and Br^−^ as an anion (Figure 6). The compound crystallizes in monoclinic crystal system, space group *P*2_1_/*c*. As expected, the charged and alkyl parts in the crystal are spatially separated and form-alternating 2D sheets of alkyl and charged groups. These 2D sheets assemble in the interdigitated pattern to form a 3D supramolecular structure (Figure 7). It should be noted that the alkyl tail experiences a fracture on the second carbon atom from the charged group, forming a dihedral angle of 71.1(2), while a conventional imidazolium surfactant with a hexadecyl tail (IA-16) does not experience a tail fracture [69]. In addition, the head group of the MPI-12 (due to the methoxyphenyl moiety) is significantly larger than that of the IA-16: length × width is 1.26 × 0.64 and 7.3 × 6.3, respectively.

**Crystal Data** for C_22_H_35_BrN_2_O (*M* = 423.43 g/mol): monoclinic, space group P2_1_/c (no. 14), *a* = 20.6305(10) Å, *b* = 14.6727(6) Å, *c* = 7.3576(3) Å, *β* = 100.176(4)°, *V* = 2192.15(17) Å^3^, *Z* = 4, *T* = 100.00(10) K, μ(Cu Kα) = 2.637 mm^−1^, *Dcalc* = 1.283 g/cm^3^, 26,691 reflections measured (4.352° ≤ 2Θ ≤ 152.982°), 4494 unique (*R*_int_ = 0.0521, R_sigma_ = 0.0310) which were used in all calculations. The final *R*_1_ was 0.0305 (I > 2σ(I)) and *wR*_2_ was 0.0797 (all data). CCDC refcode: 2207430.

It can be assumed that the sterically hindered head group in MPI-n is the main reason for the different behavior of amphiphiles (MPI-n and IA-n) upon interaction with nucleotide units. Amphiphiles with a non-functionalized imidazolium head group (which has a planar structure) are probably able to integrate more deeply between the nitrogenous bases of nucleotides without interacting with phosphate groups (therefore, there is no recharging in the systems). The steric hindrance of MPI-n does not allow amphiphiles to completely submerge between nucleotide units. Only the incorporation of the hydrophobic MPI-n tails into the nucleotide structure occurs, while the head group of the surfactant remains on the surface and is available for electrostatic binding with the phosphate anions of nucleic acids.

### 3.5. Transmission Electron Microscopy

**Figure 8:**
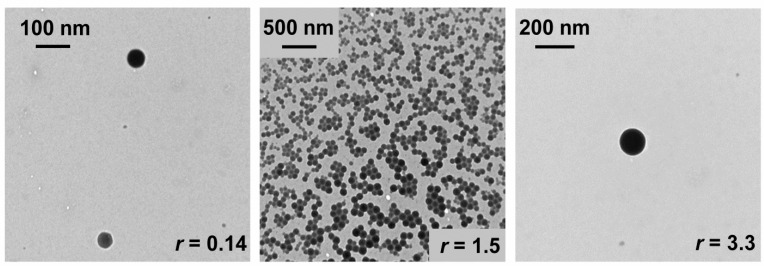
Morphology of the MPI-12/ONu lipoplexes determined by TEM.

Transmission electron microscopy (TEM) was used to determine the morphology of lipoplexes using the MPI-12/ONu system as an example. The images were obtained at different ratios of the components (*r*). Moreover, the ratios were chosen in such way as to fix complexes with a negative charge, near the isoelectric point, and with a positive charge. Figure 8 shows the MPI-12/ONu systems at *r* = 0.14, *r* = 1.5, *r* = 3.3. It can be seen that the MPI-12/ONu systems are characterized by the formation of spherical complexes. The sizes of lipoplexes at *r* = 0.14 are at the level of 80–100 nm and at *r* = 1.5 at the level of 100 nm, but sticking of particles is noticeable, which is probably due to the almost zero charge of the complexes, and at *r* = 3.3 at the level of 150 nm. The data obtained by the TEM fully correspond to the DLS data.

### 3.6. Ethidium Bromide (EB) Exclusion Assay for Surfactant/ONu and Surfactant/DNA Complexes

The ability to bind surfactants to the ONu and DNA was studied by fluorescence spectroscopy using ethidium bromide as a probe. Ethidium bromide is a cationic dye and is used to study native DNA. The ethidium ion shows a sharp increase in fluorescence intensity upon intercalation into nucleotide units. Titration of the EB/nucleic acid system with cationic surfactants results in displacement of intercalated EB from EB/nucleic acid complexes and causes fluorescence quenching [70,71]. Figure S2 shows the fluorescence spectra of free EB, the ONu/EB complex, and the ONu/EB/surfactant system. When each of four surfactants (MPI-10, MPI-12, MPI-14, MPI-16) were added to the EB/ONu complexes, smooth fluorescence quenching with complete displacement of EB was observed (as evidenced by the quenching of the EB/ONu/surfactant ternary complex fluorescence compared to the spectrum of free EB).

Moreover, the hydrocarbon tail length had a significant effect on the quenching rate: the longer the hydrocarbon tail, the faster the quenching occurred. This is probably due to the fact that hydrophobic interactions between the planar EB molecule and surfactant hydrocarbon tails contribute to the exclusion of EB from the nucleotide units [71]. The exclusion of EB may indicate the process of intercalation of surfactant between the nitrogenous bases of ONu, due to hydrophobic surfactant tails. The authors of [23] showed that when cationic surfactants are added to EB/DNA mixtures, electrostatic binding of positively charged surfactant head groups to DNA phosphate backbone occurs at the first stage. This reduces the repulsive force of negative DNA charges and makes the skeleton less rigid. An increase in the surfactant concentration can lead to the formation of micelles and quasi-micelles due to hydrophobic interaction, which leads to DNA compaction and exclusion of EB. Additionally, at the last stage, hydrophobic interactions begin to dominate.

**Figure 9:**
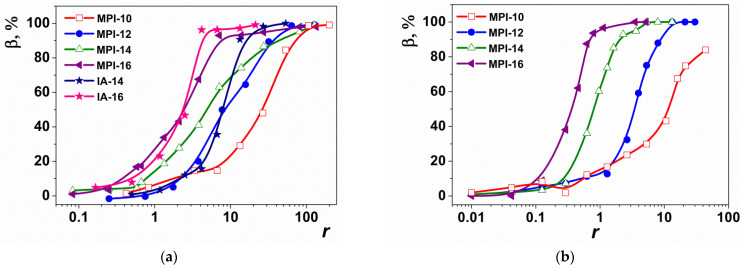
Binding degree of MPI-n with ONu (**a**) and MPI-n with DNA; (**b**) versus molar ratio of MPI-n/ONu (*r*); 25 °C.

On the one hand, it could be assumed that for the ONu/EB/MPI-n systems, EB exclusion occurs in the same way in two stages (electrostatic binding and then displacement due to hydrophobic tails). However, the calculation of the binding degree in the MPI-n/ONu systems (Figure 9a), based on fluorescence data, suggests otherwise. Figure 9a shows graphs of the binding degree of ONu to MPI-n. For comparison, a graph of the binding degree of ONu to non-functionalized imidazolium surfactants (IA-14 and IA-16) is also shown [40,41]. It can be seen that the binding isotherm for the MPI-n/ONu and IA-n/ONu systems is very similar, despite the fact that for the IA-n/ONu systems, no electrostatic binding has been recorded [40,41]. Therefore, different mechanisms can contribute to the ONu-imidazolium surfactant complexation, which can be controlled by functionalization of the head group. Probably, IA-n amphiphiles, due to their planar structure, have a higher ability to intercalate between the nitrogenous bases of nucleotides without electrostatic interaction with phosphate groups (therefore, there is no recharging in the systems). The presence of a bulky methoxyphenyl fragment in MPI-n does not allow amphiphiles to completely immerse between nucleotide units, so that the head group of the surfactant remains available for electrostatic binding with phosphate anions of nucleic acids. It should be noted that for all MPI-n/ONu complexes, the binding degree (*β*) reaches almost 100%, which is probably due to the ability of imidazolium rings to intercalate between nitrogenous bases of ONu [41,45]. The high *β* value makes it possible to recommend MPI-n surfactants as effective carriers of nucleic acids.

The binding capacity of MPI-n amphiphiles to plasmid DNA pK18 was also quantified by the fluorescence method. The fluorescence spectra recorded for EB at various surfactant/DNA molar ratios are shown in Figure S3. These data were used to calculate the binding degree (*β*) of the components. It can be seen that for higher homologues, it was possible to achieve the maximum binding degree of the components at the level of 100%, and for the amphiphile with decyl chain, the maximum binding degree to DNA was 85% (Figure 9b). As in surfactant/ONu systems, for complexes with DNA, the length of the surfactant hydrocarbon tail had a significant effect on the rate of EB quenching and, consequently, on the degree of component binding. This is due to the fact that hydrophobic interactions between the EB molecule and hydrocarbon tails of amphiphiles contribute to the displacement of EB from DNA nucleotide units [71]. Unlike ONu, the plasmid DNA pK18 molecule has a supercoiled structure; therefore, the decyl tail is probably insufficient for effective intercalation of amphiphile molecules between DNA nitrogenous bases. In this regard, *β* for MPI-10/DNA complex has a lower value.

### 3.7. Circular Dichroism Studies

**Figure 10:**
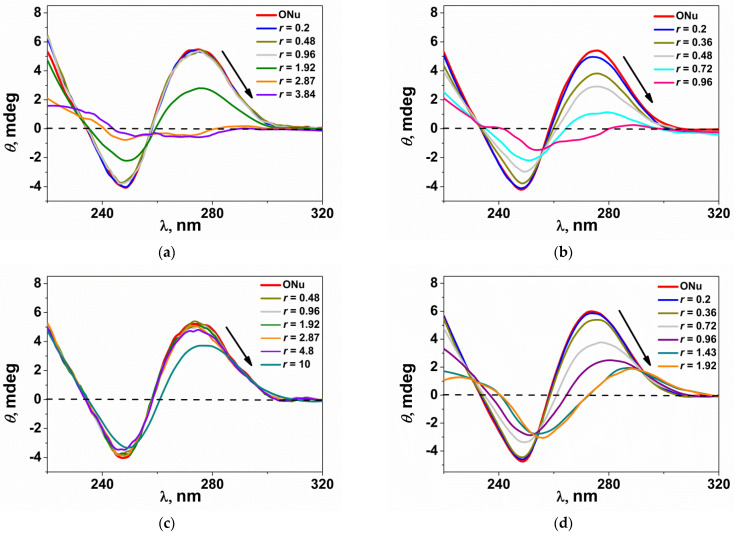
CD spectra of oligonucleotides in the presence of varying amounts of (**a**) MPI-12, (**b**) MPI-16, (**c**) IA-12, (**d**) IA-16; phosphate buffer (pH 7.4). The oligonucleotide concentration was 50 μM.

For a more detailed study of the processes occurring in the MPI-n/ONu and MPI-n/DNA systems, the method of circular dichroism spectroscopy was also used. CD spectra are an important tool for diagnosing conformational changes in DNA, since they are sensitive to changes in the secondary structure of a macromolecule [72,73,74]. Therefore, CD spectra can be useful for detecting possible structural changes associated with the formation of surfactant/ONu or surfactant/DNA complexes [75]. In particular, during the interaction of a surfactant with an oligo- and polynucleotide, conformational changes can be assessed by changes in the position and intensity of the bands in the CD spectra. Figure 10 shows the CD spectra for the oligonucleotide in the absence and presence of cationic surfactants at different N/P ratios. The experiments were carried out for amphiphiles with dodecyl and hexadecyl homologues (MPI-12, MPI-16 and their non-functionalized analogues IA-12, IA-16). The individual oligonucleotide shows a CD spectrum corresponding to conformation B, with a positive band at 275 nm due to stacking interactions between base pairs and a negative band at 245 nm due to right-handed DNA helicity [73]. In all cases, the addition of a surfactant leads to a change in the intensity and a bathochromic shift of both positive and negative bands, which is evidence of interactions in the systems. Moreover, these interactions are different for each of the homologous series. For amphiphiles with a methoxyphenyl fragment (MPI-12 and MPI-16), the intensity in both bands changes significantly, but without a marked shift observed (no more than 5 nm, Figure 10a,b). This indicates that the intercalation mechanism is partially involved in the formation of the MPI-n/ONu complexes [73,76,77] because simple groove binding and electrostatic interaction of small molecules with DNA show little or no changes in the position of positive and negative bands [78]. In the case of non-functionalized imidazolium surfactants (IA-12 and IA-16), the change in the intensity of the CD spectra in both bands is less pronounced than in MPI-n, but a significant bathochromic shift is observed with increasing *r* (up to 15 nm, Figure 10c,d). This indicates that the intercalation mechanism is more pronounced during the formation of the IA-n/ONu complexes than for the MPI-n/ONu complexes. The data obtained are consistent with the results of other methods.

### 3.8. Cytotoxicity Assay of MPI-n/ONu and MPI-n/DNA Complexes In Vitro

Further studies aimed at evaluating the cytotoxic activity of the MPI-n/ONu and MPI-n/DNA complexes in vitro. The analysis was performed on tumor cell lines of cervical cancer (M-HeLa), lung adenocarcinoma (A549), and on human normal liver cells (Chang liver). The cytotoxic effect of the complexes on selected cell lines was determined using the MTT dye reduction assay. The MTT test is a colorimetric assay used to measure cytotoxicity or cell viability [79]. Table 1 shows the IC_50_ values for the MPI-n/ONu (*r* = 1) and MPI-n/pK18 complexes (*r* = 3). It was shown that MPI-n/ONu complexes have a cytotoxic effect toward all cell lines. However, the MPI-10/ONu, MPI-14/ONu, and MPI-16/ONu systems do not show a selective effect on cancer cells. In addition, the MPI-14/ONu and MPI-16/ONu complexes are more cytotoxic toward normal Chang liver cells than toward A549 lung adenocarcinoma cancer cells. The MPI-12/ONu system turned out to be the most effective composition that showed selectivity against cancer cells. Moreover, the system acted more selectively on M-HeLa cancer cells (IC_50_ = 4.8 µM) than on A549 (IC_50_ = 7.1 µM). It should be noted that, in some cases, the use of surfactant/ONu complexes can significantly reduce the effective cytotoxic concentration compared with individual surfactant systems [47]. For example, for individual MPI-12, the IC_50_ is 18 µM toward M-HeLa and 23.7 µM toward A549, while in the complex, this concentration decreases to 4.8 µM and 7.1 µM, respectively. At the same time, the IC_50_ for normal cell lines differs slightly.

MPI-n/pK18 complexes showed a significant cytotoxic effect toward all cell lines, with selectivity for M-HeLa cancer cells. It is worth noting that mixed surfactant/DNA systems exhibited a cytotoxic effect at lower concentrations than surfactant/ONu systems and individual surfactants (Table 1). For example, the transition from MPI-10/ONu to MPI-10/DNA reduces IC_50_ by one order of magnitude (from 30 μM to 3.2 μM). In other systems, this difference is less significant and equals 1.5–2 times. However, it should be noted that the cytotoxic effect on normal liver cells is also enhanced, which can be attributed to negative phenomena.

The cytotoxicity of surfactant/DNA and surfactant/ONu complexes—in particular, the phenomenon of decreasing cytotoxicity of the systems—depends on many parameters, such as the charge ratio between the cationic lipid and the nucleic acid (N/P), the type of composition, the dose of the administered complexes, the incubation time, the type and density of cells, and the morphology of lipoplexes [80,81]. In general, lipoplexes with higher charge ratios of N/P are generally more toxic to various cell types, including cancer cell lines [80,81]. In addition, in the works [82,83], it was established that mixed-composition cationic lipids/DNA enhance cytotoxicity. Free DNA was shown to have no cytotoxic effect, and cationic lipids caused only a small amount of cellular toxicity. However, lipoplexes induced significant cytotoxicity against a wide range of cancer cells compared to the individual components. Another study showed that the cytotoxicity of complexes with DNA may depend on the nature of the aggregates formed [84]. For example, it was found that the same lipoplex showed significantly reduced toxicity in a vesicular form, compared with micellar. In the systems under study, the difference in cytotoxicity may be due to different N/P ratios in the surfactant/DNA (*r* = 3) and surfactant/ONu (*r* = 1) complexes, the type of genetic material, as well as various morphology of complexes.

### 3.9. Flow Cytometry Assay

**Figure 11:**
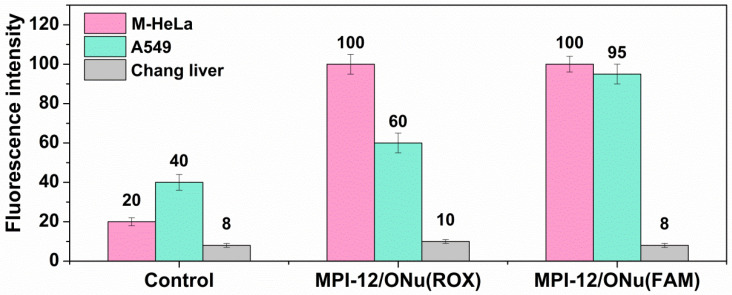
Cellular uptake study of MPI-12/ONu(ROX) and MPI-12/ONu(FAM) complexes by M-HeLa, A549 cancer cells and Chang liver normal cell lines.

As shown, the MPI-12/ONu system was most beneficial in terms of cytotoxicity studies. Therefore, further experiments were focused on this system. The ability of the complex internalization with cancer and normal cells was assessed by flow cytometry. Since flow cytometry is based on light scattering and fluorescence signals, two types of oligonucleotides of the same sequence as in previous experiments, including fluorescent labels, were used for the experiments. The fluorescent labels of carboxy-X-rhodamine (ONu(ROX)) and carboxyfluorescein (ONu(FAM)) were included in the selected oligonucleotides. Complexes containing ONu(ROX) showed red fluorescence and complexes containing ONu(FAM) showed green fluorescence. Cellular uptake of MPI-12/ONu(ROX) and MPI-12/ONu(FAM) by M-HeLa and A549 cancer cells, as well as by normal Chang liver cells, was assessed at concentrations corresponding to IC_50_/2 (Figure 11).

It can be seen that 24 h treatment of M-HeLa cells with MPI-12/ONu(ROX) complex resulted in a significant increase in the average fluorescence intensity compared to the control. However, the same system on A549 cells caused a lesser extent of the fluorescence intensity increase. The study of the MPI-12/ONu(FAM) complex showed that they also effectively penetrated into M-HeLa and A549 cancer cells, but penetration into A549 cells was worse. At the same time, the internalization of both systems into normal Chang liver cells was very low; almost at the control level. Probably, the difference in the penetration of surfactant/ONu complexes into M-HeLa and A549 cancer cells is due to the different mechanisms of interaction of these systems with cell membranes. Indeed, the degree of interaction of lipoplexes with cell surfaces is significantly influenced by cellular parameters. It has been established that the efficiency of transfection depends on the type of cells, which may differ in cell cycle, endocytic capacity, as well as the frequency of cell divisions [85,86].

### 3.10. Fluorescence Microscopy

**Figure 12:**
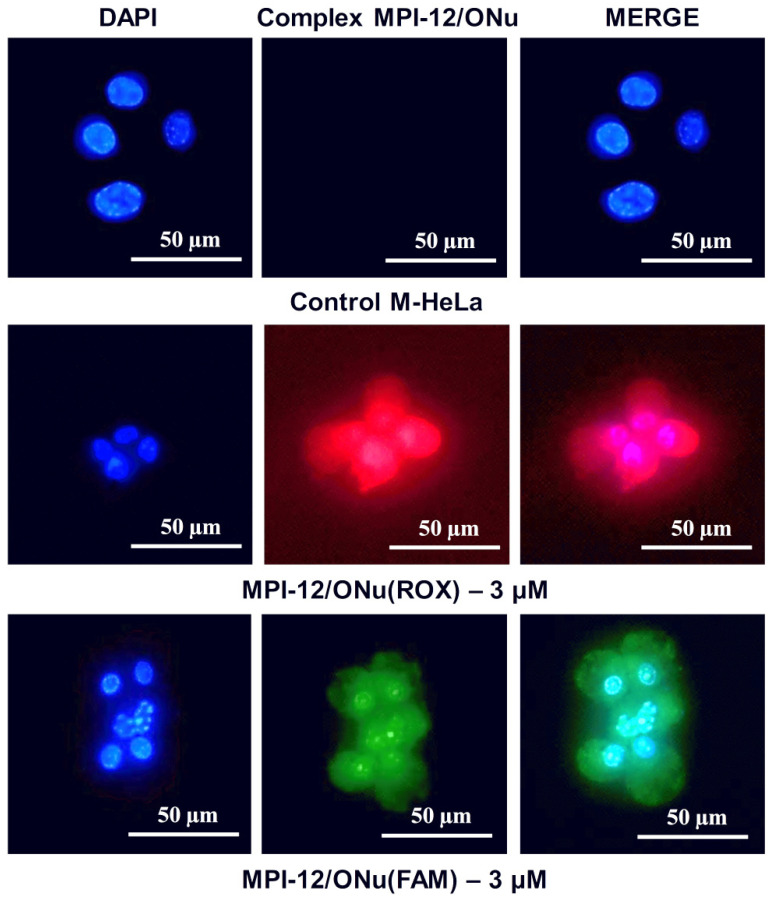
Qualitative analysis of uptake study of MPI-12/ONu(ROX) and MPI-12/ONu(FAM) complexes by M-HeLa cells using the fluorescence microscopy. Complexes containing ONu(ROX) showed red fluorescence and complexes containing ONu(FAM) showed green fluorescence.

For better understanding of the mechanism of action of surfactant/ONu complexes on cancer and normal cells, the fluorescence microscopy was further used. This study focused on the location of the MPI-12/ONu(ROX) and MPI-12/ONu(FAM) systems inside the M-HeLa, A549, and Chang liver cells. The cell nuclei were stained with DNA intercalating dye DAPI (blue fluorescence). Complexes containing ONu(ROX) showed red fluorescence and complexes containing ONu(FAM) showed green fluorescence. Figure 9, Figure 10 and Figure 11 show the results of internalization of the MPI-12/ONu(ROX) and MPI-12/ONu(FAM) complexes into cells of different nature. The data obtained correspond to those obtained via flow cytometry. The MPI-12/ONu(ROX) and MPI-12/ONu(FAM) complexes most efficiently penetrated into M-HeLa cells. During the experiment, intense red fluorescence (MPI-12/ONu(ROX)) or green fluorescence (MPI-12/ONu(FAM)) was observed in the cytoplasm and nuclei of cells (purple and blue–green spots on the combined images) (Figure 12).

**Figure 13:**
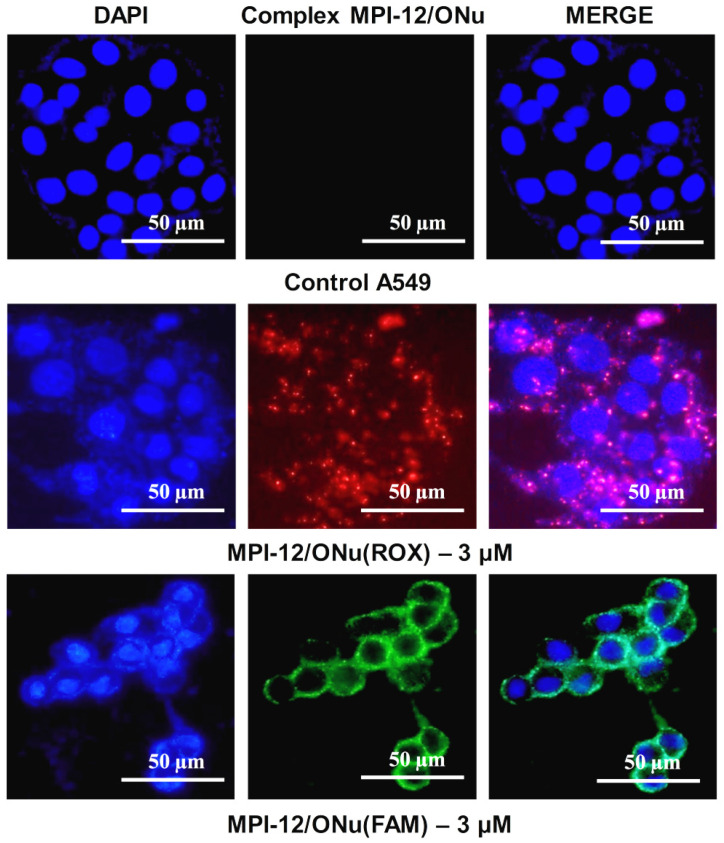
Qualitative analysis of uptake study of MPI-12/ONu(ROX) and MPI-12/ONu(FAM) complexes by A549 cells using the fluorescence microscopy. Complexes containing ONu(ROX) showed red fluorescence and complexes containing ONu(FAM) showed green fluorescence.

It was also shown that in the case of treatment of the lung adenocarcinoma (A549) cells with MPI-12/ONu(ROX) and MPI-12/ONu(FAM) lipoplexes, less intense luminescence was observed (Figure 13). The complexes were localized mainly in the cytoplasm of cells and on the surface of nuclear membranes, but no lipoplex fluorescence was observed in cell nuclei. These results indirectly confirm that for the MPI-12/ONu(ROX) and MPI-12/ONu(FAM) complexes, internalization into M-HeLa and A459 cancer cells occurs via different pathways. It is generally accepted that the main mechanism of internalization of lipoplexes into cells is the endocytic pathway [87], which is further subdivided into four types: clathrin-mediated endocytosis, phagocytosis, caveolar-mediated endocytosis, and macropinocytosis [87,88]. The type of endocytic pathway is affected by a number of factors; for example, the physicochemical properties of the nanocarrier (size, surface charge, presence of a specific ligand), as well as the type of cell line [87]. As a rule, internalization by endocytic pathways involves the uptake of complexes with DNA by intracellular vesicles and fusion with lysosomes [87], i.e., all endocytic pathways lead to the pathway of the endolysosomal system [89]. Therefore, endosomal escape is a significant factor influencing the success of non-viral delivery of nucleic acids. Along with this, transcription of foreign DNA in the cell requires delivery to the nucleus. The main pathway of foreign DNA penetration into the cell nucleus is passive capture during mitosis [89]. At this moment, the nuclear envelope is destroyed and its components (proteins and membranes) are associated with the endoplasmic reticulum [90]. Therefore, the delivery of nucleic acids released from non-viral vectors to dividing cells depends on the amount of intact DNA present near chromatin at the time of cell division. Mitosis-dependent cell transfection can be significantly limited in cell lines with infrequent cell division [89]. Therefore, it can be assumed that in our case, it is the difference in the structure of cell lines that is the main reason for the different internalizations of the MPI-12/ONu(ROX) and MPI-12/ONu(FAM) complexes in cancer cells.

**Figure 14:**
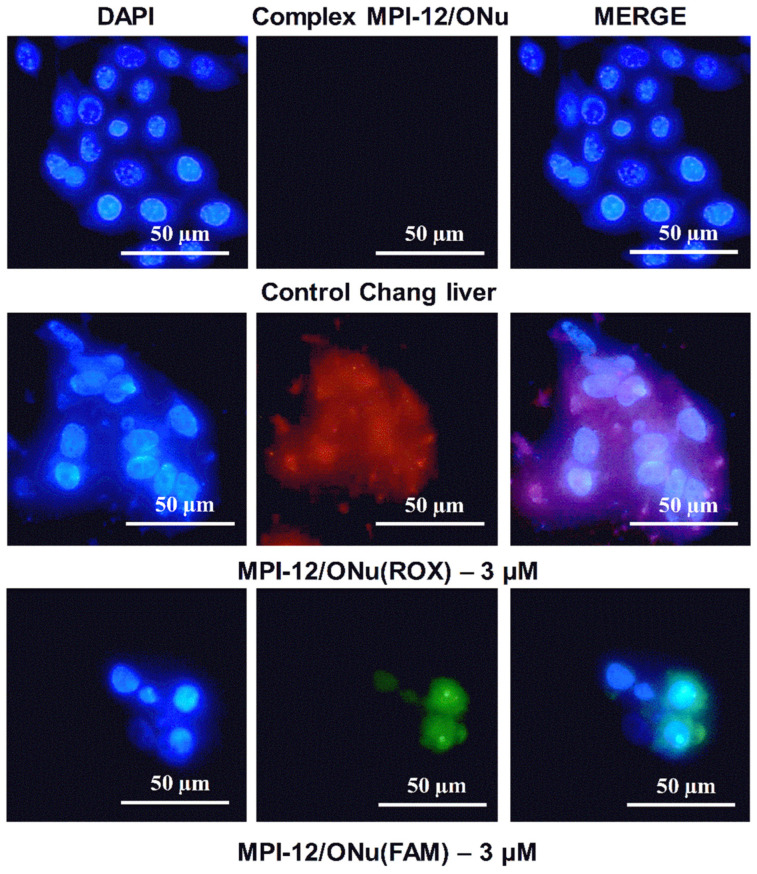
Qualitative analysis of cellular uptake study of MPI-12/ONu(ROX) and MPI-12/ONu(FAM) complexes by Chang liver cells using the fluorescence microscopy. Complexes containing ONu(ROX) showed red fluorescence and complexes containing ONu(FAM) showed green fluorescence.

In case of treatment of healthy Chang liver cells with complexes (MPI-12/ONu(ROX) and MPI-12/ONu(FAM)) as in flow cytometry experiments, very low fluorescence intensity occurred (Figure 14) especially for MPI-12/ONu(FAM). However, superposition of two images taken at different excitation wavelengths showed that lipoplexes are localized both in the nucleus and in the cytoplasm of the cells. The weak fluorescence intensity is probably due to the fact that the MPI-12/ONu(ROX) and MPI-12/ONu(FAM) complexes accumulate better in tumor cells (due to the effect of increased permeability and retention) than in normal cells (due to hypervascularization) [91,92].

### 3.11. In Vitro Transfection Studies into M-HeLa and A549 Cell Lines

**Figure 15:**
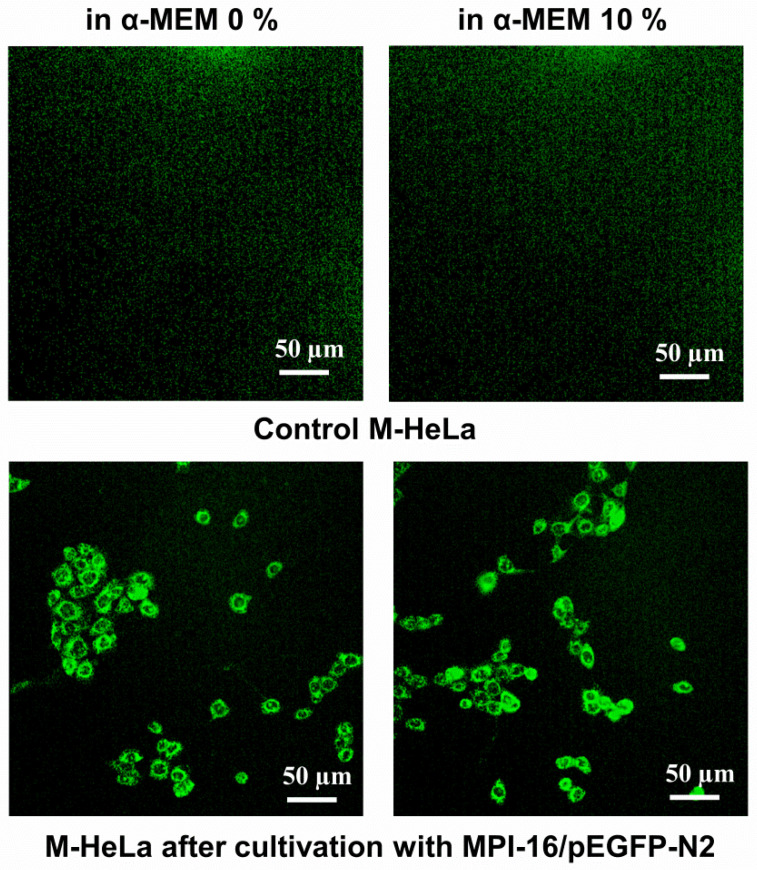
Representative fluorescent microphotographs of M-HeLa cells transfected with MPI-16/pEGFP-N2 complexes under different conditions: 0% α-MEM and 10% α-MEM; cultivation 24 h.

Next, the transfection efficiency of the MPI-12/DNA and MPI-16/DNA complexes was determined by flow cytometry using M-HeLa and A549 cancer cells (Figures S4–S7). Plasmid DNA pEGFP-N2 was used as genetic material. According to flow cytometry data, the MPI-12/DNA and MPI-16/DNA complexes showed pronounced transfection activity, which is highly competitive with that of the reference preparation Lipofectamine 3000. Importantly, the efficacy of the lipoplexes strongly depends on the surfactant structure, the type of the cells, and the medium conditions. Since our work focuses on the design of surfactants capable of delivering the gene material, it is of particular interest that in serum-free medium, the expression of the target gene for MPI-16 compared to MPI-12 was 3.4-fold (A549) and 2-fold (M-HeLa) higher, with a higher level of expression in M-HeLa cells (Table 2). Representative fluorescence microphotographs of the cells transfected with MPI-16/pEGFP-N2 complexes under different conditions are shown in Figure 15 and Figure S8.

It should be noted that the complexes of cationic surfactants with pDNA, when added to the culture medium, additionally aggregated, forming a precipitate, especially in the case of MPI-16. It can be assumed that the increased transfection activity of surfactants is associated with the formation of their aggregates with incorporated pDNA, which are presumably formed even in the absence of a clear DNA-binding ability (according to electrophoretic analysis for MPI-16). These aggregates can be probably captured by the cells and contribute to intracellular pDNA pharmacokinetics (for example, due to release from lysosomes, protection from degradation, and other mechanisms). Serum proteins seem to somehow influence the formation of aggregates or their interaction with cells, thereby inhibiting the efficiency of transfection.

It should be emphasized that this study demonstrated encouraging results with regard to the gene delivery potential of the MPI surfactants. At the same time, further efforts are needed to elucidate factors controlling the transfection efficacy and the mechanisms contributing to the MPI surfactants/DNA interaction, which will require a separate study.

### 3.12. Hemagglutination Analysis for MPI/ONu Complexes

**Figure 16:**
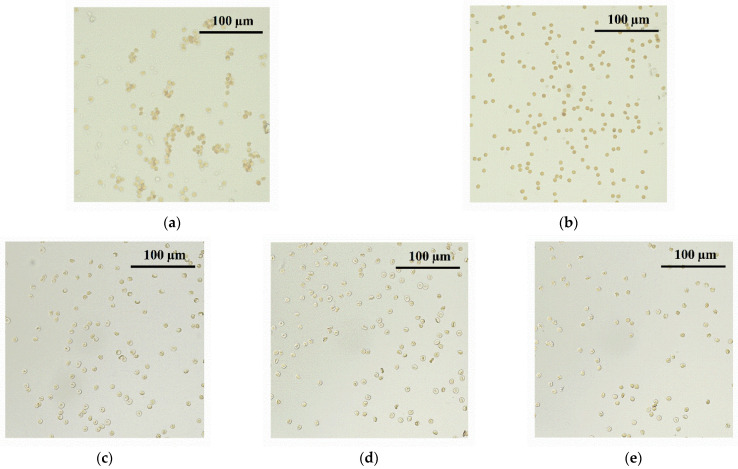
The effect of MPI-12/ONu complexes on erythrocyte agglutination was observed by bright-field microscopy: (**a**) in a mixture of type A (II) and B (III) erythrocytes—positive control of agglutination; (**b**) negative control of agglutination; (**c**) the effect of MPI-12/ONu complexes on erythrocyte agglutination at *r* = 0.6 (molar ratio of MPI-12/ONu = 0.034/0.056); (**d**) the effect of MPI-12/ONu complexes on erythrocyte agglutination at *r* = 1 (molar ratio of MPI-12/ONu = 0.028/0.028); (**e**) the effect of MPI-12/ONu complexes on erythrocyte agglutination at *r* = 2 (molar ratio of MPI-12/ONu = 0.028/0.056).

In vitro physicochemical and biological studies of complexes with DNA (or ONu) can show very promising results for a potential use of non-viral vectors. However, the successful functioning of a non-viral vector in vivo does not solely depend on the physicochemical parameters and composition of the carrier. To improve gene delivery in vivo, it is necessary to understand the interaction of DNA complexes with blood components. It should be mentioned that the interaction of lipoplexes with blood plasma proteins is studied to some extent [93,94,95], but there is little information on the interaction of lipoplexes with blood erythrocytes. Therefore, hemagglutination in the presence of the MPI-n/ONu complexes was assessed. The experiments were carried out on the example of the MPI-12/ONu system. Hemagglutination is the process of agglutination and sedimentation of erythrocytes under the influence of agents (agglutinins) that can be adsorbed on the surface of erythrocytes. The cytoplasmic membrane of erythrocytes consists of substances of a protein nature—agglutinogens—and nanocontainers can act as agglutinins. As a rule, agglutination of erythrocytes is directly related to the zeta potential of nanocontainers [96], so the study of hemagglutination was carried out at different molar ratio of N/P (*r* = 0.6; *r* = 1; *r* = 2). The results of the hemagglutination activity assessment are presented in Figure S9 and Table 3. The negative control shows a round red button, which corresponds to normal erythrocytes in saline (Figure S9, Negative control). With erythrocyte agglutination, a cloudy red suspension is observed (Figure S9, positive control). It can be seen that the MPI-12/ONu complexes cause an agglutinating effect only at high concentrations. It should be noted that agglutination for the MPI-12/ONu complexes begins at concentrations that are significantly higher than the effective cytotoxic concentrations. Therefore, these systems can be used in vivo at certain concentrations. Additionally, for all systems, erythrocytes were shown by fluorescent microscopy at concentrations of MPI-12/ONu complexes in which there was no agglutination (Figure 16).

## 4. Conclusions

Lipoplexes based on imidazolium surfactants with methoxyphenyl fragment and nucleic acids of various types (oligonucleotide and plasmid DNA pK18) were obtained and studied using various methods. The formation of mixed complexes of optimal size (about 100–200 nm) with the participation of electrostatic, hydrophobic interactions, and intercalation mechanism was shown. It has been established that hydrocarbon tail length has a significant effect on the surfactant/nucleic acid interaction: the higher tendency of amphiphiles to self-organize makes complexation more favorable. The study of the cytotoxic effect of the complexes against M-HeLa and A549 cancer cells and Chang liver normal cells revealed that the transition from surfactant/ONu to surfactant/DNA complexes is accompanied by a decrease in the cytotoxic concentrations up to 10 times. The selective cytotoxic effect of the complexes toward M-HeLa cancer cells and the high ability of the systems to be transfected into cancer cells were shown. Moreover, depending on the type of cell line, the mechanism of internalization of complexes into cells probably differs. Surfactant/DNA complexes showed a pronounced transfection activity that is highly competitive with that of the referenced preparation, Lipofectamine 3000, which depends on the surfactant structure, the type of the cells, and the medium conditions. Thus, lipoplexes based on amphiphile with dodecyl tail penetrate into the cytoplasm and nuclei of M-HeLa cells, while in A549 cells, they are found only in the cytoplasm. The results obtained show that imidazolium surfactants with methoxyphenyl fragment are effective agents for compacting nucleic acids and can be effective non-viral vectors. The results of this work provide further insight into the key parameters that control complexation in surfactant/nucleic acid systems, which is of relevance for the targeted design of lipoplexes for gene therapy.

## Figures and Tables

**Table 1:** The cytotoxic effect of MPI-n amphiphiles, MPI-n/ONu and MPI-n/pK18 binary systems toward normal and tumor human cell lines (surfactant/ONu molar ratio is 1:1; surfactant/DNA molar ratio is 3:1).

Compounds	IC_50_ (µM)		
	Cancer Cell Lines		Normal Cell Line
	^a^ M-HeLa	^b^ A549	^c^ Chang Liver
MPI-10/ONu	30 ± 2.3	21.0 ± 1.8	28.7 ± 2.3
MPI-12/ONu	4.8 ± 0.4	7.1 ± 0.6	10.6 ± 0.8
MPI-14/ONu	6.2 ± 0.5	10.7 ± 0.8	7.9 ± 0.6
MPI-16/ONu	5.1 ± 0.4	14.8 ± 1.2	7.2 ± 0.6
MPI-10/pK18	3.2 ± 0.3	9.4 ± 0.7	8.4 ± 0.7
MPI-12/pK18	4.4 ± 0.3	5.8 ± 0.5	7.0 ± 0.6
MPI-14/pK18	3.0 ± 0.2	5.8 ± 0.5	7.0 ± 0.6
MPI-16/pK18	3.1 ± 0.1	5.0 ± 0.4	8.7 ± 0.7
MPI-10 [47]	28.3 ± 2.2	22.1 ± 1.7	26.9 ± 2.1
MPI-12 [47]	18.0 ± 1.4	23.7 ± 1.9	13.2 ± 1.1
MPI-14 [47]	6.3 ± 0.5	22.4 ± 1.7	10.0 ± 0.9
MPI-16 [47]	11.5 ± 0.9	27.8 ± 2.2	13.1 ± 1.2

^a^ M-HeLa is a human cervix epitheloid carcinoma; ^b^ A549 is an adenocarcinomic human alveolar basal epithelial cell line; ^c^ Chang liver is normal human liver cells. The experiments were repeated three times.

**Table 2:** Mean channel fluorescence of A549 and M-HeLa cells transfected with (MPI-12(650 µg/mL)/pEGFP-N2, MPI-16(325 µg/mL)/pEGFP-N2).

Compounds	A549	M-HeLa
In serum-free medium (α-MEM/0% FBS)		
Lipofectamine 3000	0.055 ± 0.010	0.233 ± 0.019
MPI-12	0.486 ± 0.026	0.980 ± 0.003
MPI-16	1.650 ± 0.010	1.945 ± 0.008
In a medium with serum (α-MEM/10% FBS)		
Lipofectamine 3000	0.013 ± 0.002	0.420 ± 0.008
MPI-12	0.427 ± 0.022	0.269 ± 0.001
MPI-16	0.643 ± 0.002	0.436 ± 0.012

**Table 3:** Assessment of the ability of MPI-12/ONu complexes to hemagglutination.

	C_ONu_, mM	r = 0.6		r = 1		r = 2	
		C_MPI-12_, mM	Agglutination +/−	C_MPI-12_, mM	Agglutination +/−	C_MPI-12_, mM	Agglutination +/−
A	0.002	0.001	−	0.002	−	0.004	−
B	0.004	0.002	−	0.004	−	0.007	−
C	0.007	0.004	−	0.007	−	0.014	−
D	0.014	0.008	−	0.014	−	0.028	−
E	0.028	0.017	−	0.028	−	0.056	−
F	0.056	0.034	−	0.056	+	0.113	+
G	0.113	0.068	+	0.113	+	0.225	+
H	0.225	0.135	+	0.225	+	0.450	+

## Data Availability

CCDC 2207430 contain the supplementary crystallographic data for this paper. These data can be obtained free of charge via www.ccdc.cam.ac.uk/data_request/cif (accessed on 15 September 2022), or by emailing data_request@ccdc.cam.ac.uk, or by contacting The Cambridge Crystallographic Data Centre, 12 Union Road, Cambridge CB2 1EZ, UK; Fax: +44-1223-336033.

## Supplementary Materials

The following supporting information can be downloaded at: https://www.mdpi.com/article/10.3390/pharmaceutics14122685/s1. Table S1. The hydrodynamic diameter (D_H_, nm) of MPI-n/ONu complexes depending on the molar ratio of components (*r*). Figure S1. Number-averaged size distribution of the MPI-14/pK18 (a) and MPI-16/pK18 (b) complexes at different molar ratios; 25 °C. Figure S2. Fluorescence emission spectra of EB/ONu complexes in the presence of various amounts of MPI-n: (a) MPI-10/ONu, (b) MPI-12/ONu, (c) MPI-14/ONu, (d) MPI-16/ONu; 25 °C. Figure S3. Fluorescence emission spectra of EB/pK18 complexes in the presence of various amounts of MPI-n: (a) MPI-10/pK18, (b) MPI-12/pK18, (c) MPI-14/pK18, (d) MPI-16/pK18; 25 °C. Figure S4. Distribution of green fluorescence in A549 cells cultured in the presence of MPI-n/pEGFP-N2 complexes for 24 h: (a)—Control (untreated cells), (b)—Lipofectamine 3000/pEGFP-N2, (c)—MPI-12(650 µg/mL)/pEGFP-N2, (d)—MPI-16(325 µg/mL)/pEGFP-N2. Red bar confines transfected cells region; percentage value indicates transfection efficacy relative to total cell number. Cells were treated with 4-times diluted complexes and cultured in α-MEM 0% FBS for 4 h. Lipofectamine 3000 was applied according to the manufacturer’s protocol. Figure S5. Distribution of green fluorescence in M-HeLa cells cultured in the presence of MPI-n/pEGFP-N2 complexes for 24 h: (a)—Control (untreated cells), (b)—Lipofectamine 3000/pEGFP-N2, (c)—MPI-12(650 µg/mL)/pEGFP-N2, (d)—MPI-16(325 µg/mL)/pEGFP-N2. Red bar confines transfected cells region; percentage value indicates transfection efficacy relative to total cell number. Cells were treated with 4-times diluted complexes and cultured in α-MEM 0% FBS for 4 h. Lipofectamine 3000 was applied according to the manufacturer’s protocol. Figure S6. Distribution of green fluorescence in A549 cells cultured in the presence of MPI-n/pEGFP-N2 complexes for 24 h: (a)—Control (untreated cells), (b)—Lipofectamine 3000/pEGFP-N2, (c)—MPI-12(650 µg/mL)/pEGFP-N2, (d)—MPI-16(325 µg/mL)/pEGFP-N2. Red bar confines transfected cells region; percentage value indicates transfection efficacy relative to total cell number. Cells were treated with 4-times diluted complexes and cultured in α-MEM 10% FBS for 4 h. Lipofectamin 3000 was applied according to the manufacturer’s protocol. Figure S7. Distribution of green fluorescence in M-HeLa cells cultured in the presence of MPI-n/pEGFP-N2 complexes for 24 h: (a)—Control (untreated cells), (b)—Lipofectamine 3000/pEGFP-N2, (c)—MPI-12(650 µg/mL)/pEGFP-N2, (d)—MPI-16(325 µg/mL)/pEGFP-N2. Red bar confines transfected cells region; percentage value indicates transfection efficacy relative to total cell number. Cells were treated with 4-times diluted complexes and cultured in α-MEM 10% FBS for 4 h. Lipofectamine 3000 was applied according to the manufacturer’s protocol. Figure S8. Representative fluorescent microphotographs of A549 cells transfected with MPI-16/pEGFP-N2 complexes under different conditions: 0% α-MEM and 10% α-MEM; cultivation 24 h. Figure S9. Photograph of microplate wells containing blood samples supplemented with MPI-12/ONu complexes at various *r*.
