# Supplementary material for: Complexation of Oligo- and Polynucleotides with Methoxyphenyl-Functionalized Imidazolium Surfactants
Source: Pharmaceutics. 2022 Dec 1;14(12):2685. doi: 10.3390/pharmaceutics14122685 (PMC9782993; doi:10.3390/pharmaceutics14122685)
Supplement: Supplementary file 1 [file pharmaceutics-14-02685-s001.zip › pharmaceutics-2012235-supplementary.pdf]

pharmaceutics

Supplementary Materials

Complexation of Oligo- and Polynucleotides with Methoxyphenyl-Functionalized Imidazolium Surfactants

Darya A. Kuznetsova [1,*], Denis M. Kuznetsov [1], Leysan A. Vasileva [1], Syumbelya K. Amerhanova [1], Dilyara N. Valeeva [2], Diana V. Salakhieva [3], Viktoriia A. Nikolaeva [3], Irek R. Nizameev [1], Daut R. Islamov [4], Konstantin S. Usachev [4], Alexandra D. Voloshina [1] and Lucia Ya. Zakharova [1]

[1] Arbuzov Institute of Organic and Physical Chemistry, FRC Kazan Scientific Center, Russian Academy of Sciences, Arbuzov Str. 8, 420088 Kazan, Russia
[2] Institute of Innovation Management, Kazan National Research Technological University, Karl Marx Str. 68, 420015 Kazan, Russia
[3] Institute of Fundamental Medicine and Biology, Kazan (Volga Region) Federal University, Kremlyovskaya Str. 18, 420008 Kazan, Russia
[4] FRC Kazan Scientific Center of RAS, Russian Academy of Sciences, Lobachevsky Street 2/31, 420111 Kazan, Russia
* Correspondence: dashyna111@mail.ru

Table S1. The hydrodynamic diameter (D_H, nm) of MPI-n/ONu complexes depending on the molar ratio of components (r)

Compounds	Surfactant/ONu molar ratio (*r*)	D_H, nm
ONu	-	3.122±0.5
MPI-10/ONu	0.06	58.77±3
MPI-10/ONu	0.4	91.28±2
MPI-10/ONu	0.86	105.7±3
MPI-10/ONu	1.77	105.7±4
MPI-10/ONu	3.6	531.2±9
MPI-10/ONu	5.5	255±5
MPI-10/ONu	10	122.4±4
MPI-12/ONu	0.06	105.7±9
MPI-12/ONu	0.14	78.82±8
MPI-12/ONu	0.55	122.4±6
MPI-12/ONu	1.1	122.4±7
MPI-12/ONu	1.5	531.2±5
MPI-12/ONu	3.3	164.2±5
MPI-12/ONu	4.5	164.2±4
MPI-14/ONu	0.06	37.84±5
MPI-14/ONu	0.18	50.75±6
MPI-14/ONu	0.31	91.28±8
MPI-14/ONu	0.58	78.82±5
MPI-14/ONu	1.15	122.4±9
MPI-14/ONu	1.7	122.4±9
MPI-14/ONu	2.8	122.4±9

MPI-16/ONu	0.02	8.721±2
MPI-16/ONu	0.08	78.82±3
MPI-16/ONu	0.12	105.7±5
MPI-16/ONu	0.53	91.28±4
MPI-16/ONu	1.1	141.8±8
MPI-16/ONu	1.7	164.2±9
MPI-16/ONu	2.8	141.8±9

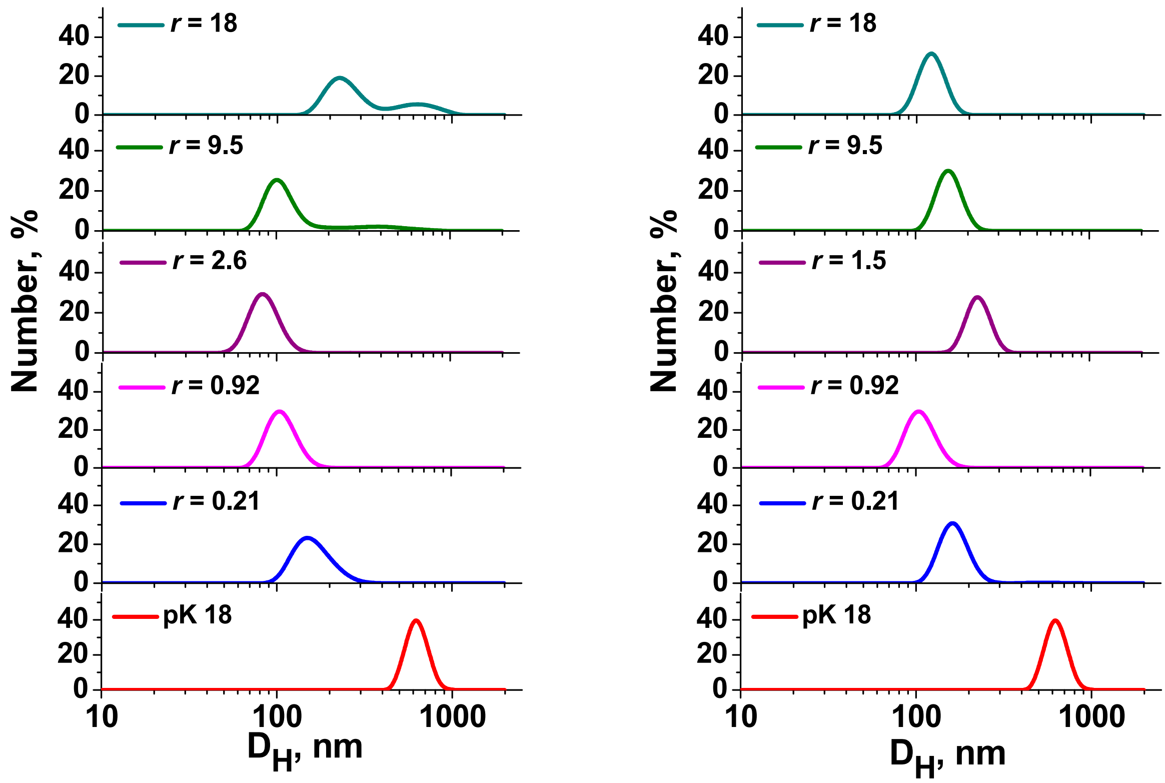

(a) (b)

Figure S1. Number-averaged size distribution of the MPI-14/pK18 (a) and MPI-16/pK18 (b) complexes at different molar ratios; 25 °C

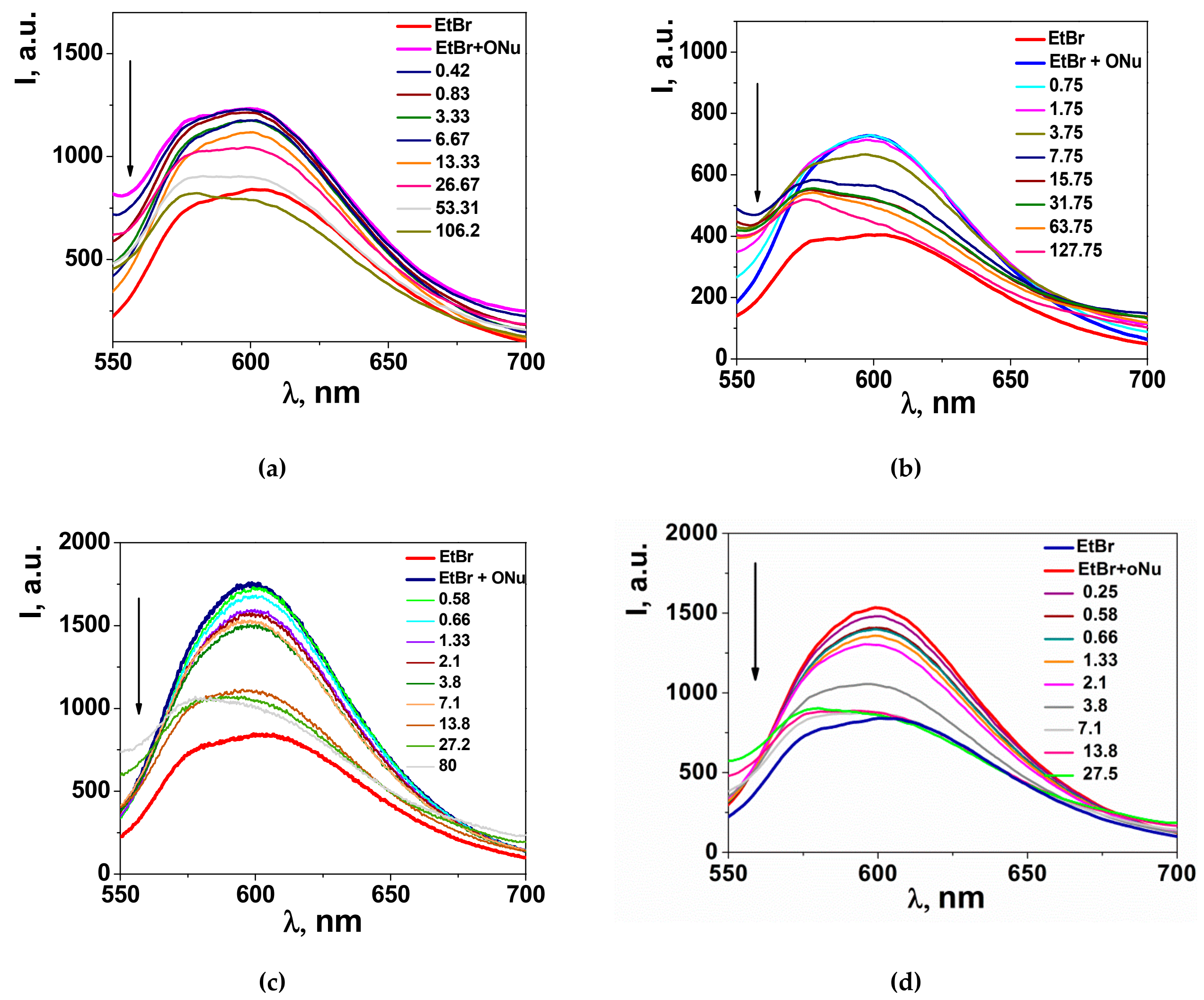

Figure S2. Fluorescence emission spectra of EB/ONu complexes in the presence of various amounts of MPI-n: (a) MPI-10/ONu, (b) MPI-12/ONu, (c) MPI-14/ONu, (d) MPI-16/ONu; 25 °C.

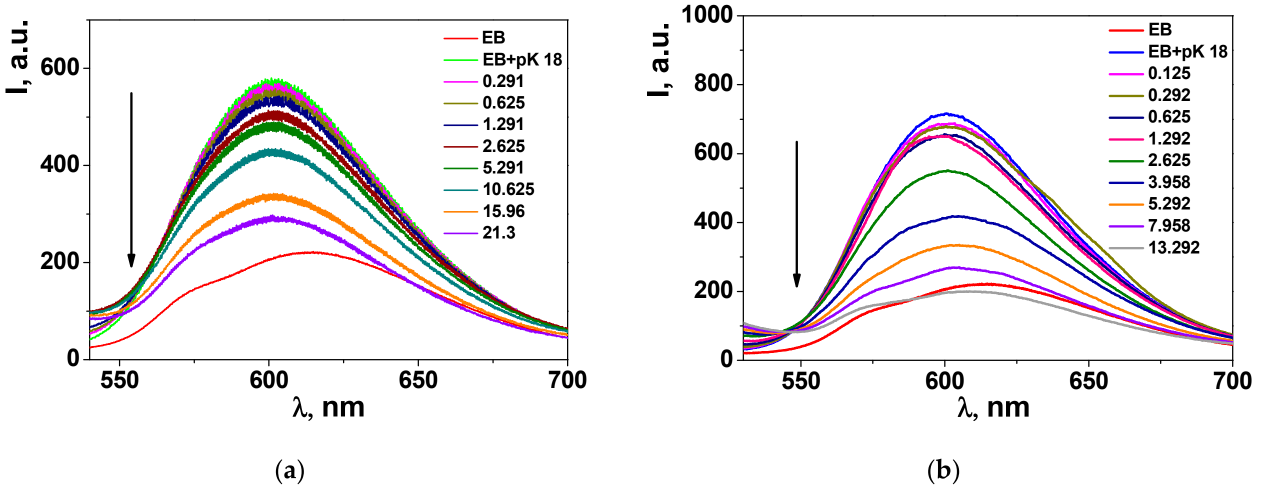

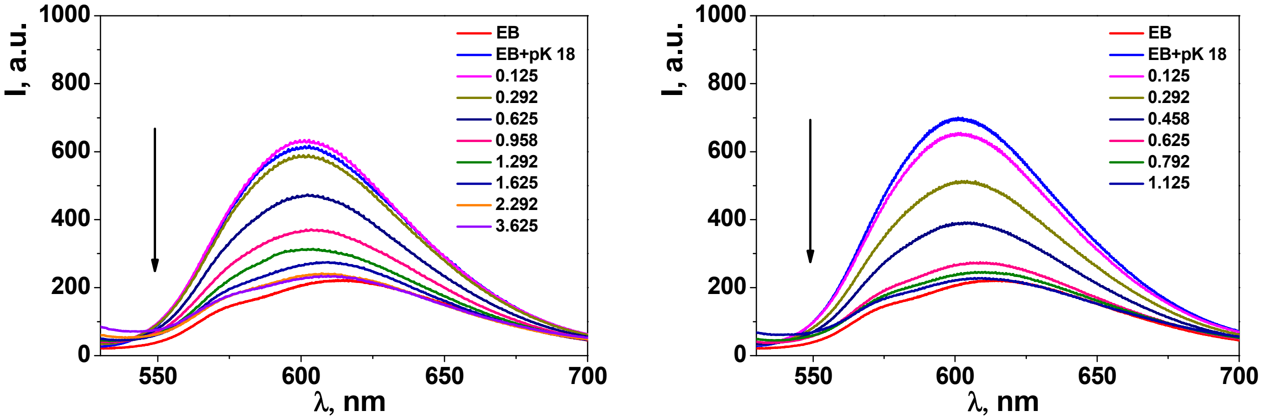

(c) (d)

Figure S3. Fluorescence emission spectra of EB/pK18 complexes in the presence of various amounts of MPI-n: (a) MPI-10/pK18, (b) MPI-12/pK18, (c) MPI-14/pK18, (d) MPI-16/pK18; 25 °C.

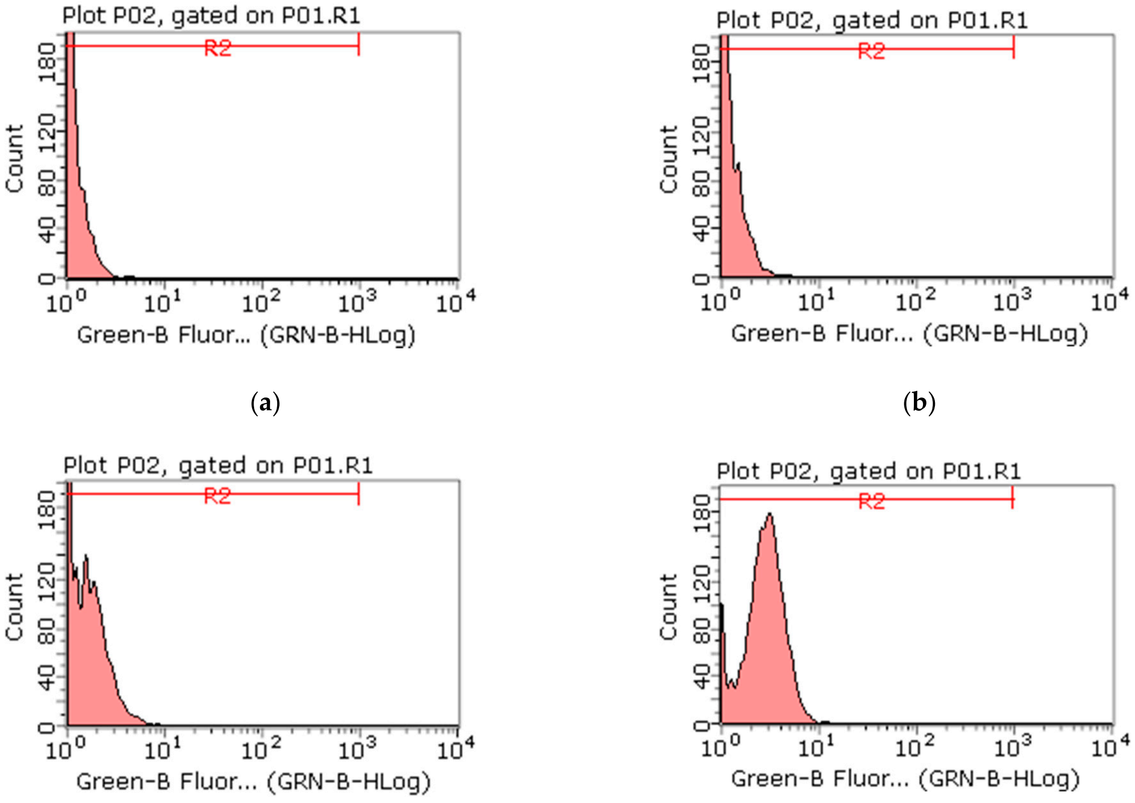

(**a**) (**b**)

(c) (d)

Figure S4. Distribution of green fluorescence in **A549** cells cultured in the presence of MPI-n/pEGFP-N2 complexes for 24 h: (a) – Control (untreated cells), (b) – Lipofectamine 3000/pEGFP-N2, (c) – MPI-12(650 μg/ml)/pEGFP-N2, (d) – MPI-16(325 μg/ml)/pEGFP-N2. Red bar confines transfected cells region; percentage value indicates transfection efficacy relative to total cell number. Cells were treated with 4-times diluted complexes and cultured in **α-MEM 0% FBS** for 4 h. Lipofectamine 3000 was applied according to the manufacturer's protocol.

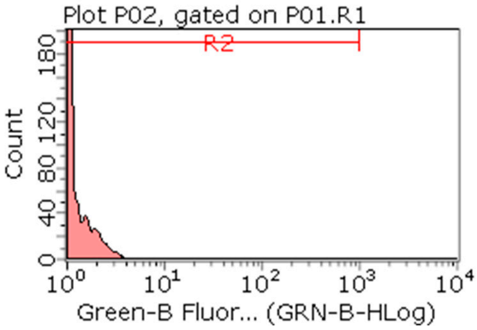

(**a**)

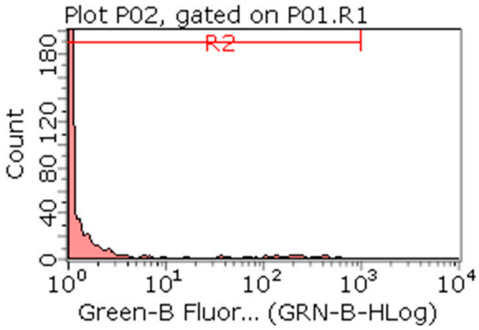

(**b**)

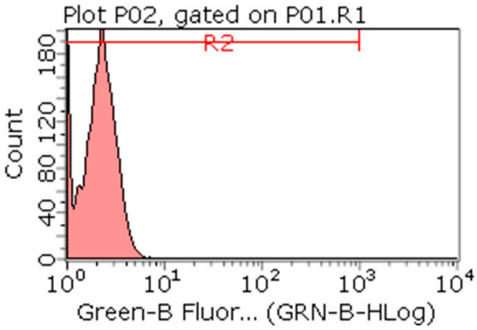

(c)

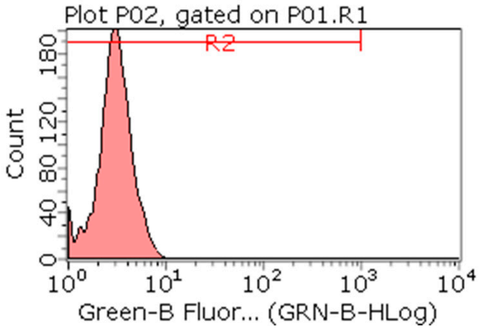

(d)

Figure S5. Distribution of green fluorescence in **M-HeLa cells** cultured in the presence of MPI-n/pEGFP-N2 complexes for 24 h: (a) – Control (untreated cells), (b) – Lipofectamine 3000/pEGFP-N2, (c) – MPI-12(650 μg/ml)/pEGFP-N2, (d) – MPI-16(325 μg/ml)/pEGFP-N2. Red bar confines transfected cells region; percentage value indicates transfection efficacy relative to total cell number. Cells were treated with 4-times diluted complexes and cultured in ***α*-MEM 0% FBS** for 4 h. Lipofectamine 3000 was applied according to the manufacturer's protocol.

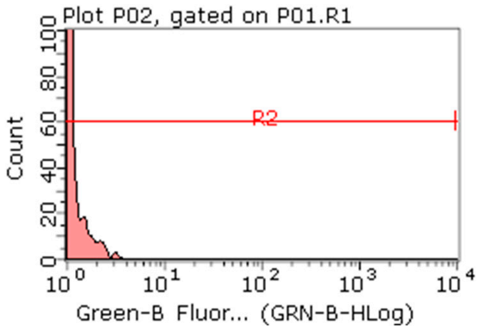

(**a**)

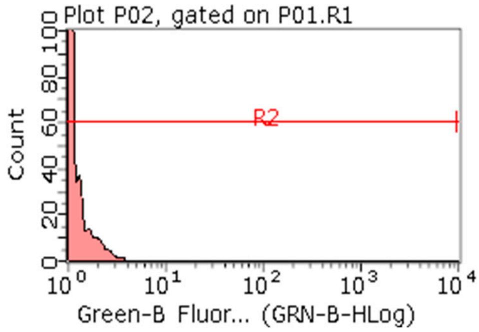

(**b**)

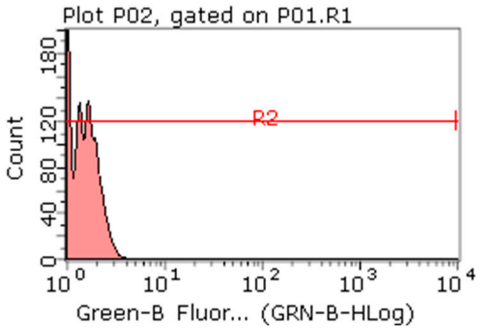

(c)

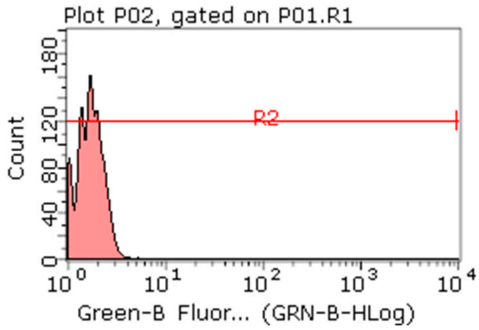

(d)

Figure S6. Figure. Distribution of green fluorescence in **A549** cells cultured in the presence of MPI-n/pEGFP-N2 complexes for 24 h: (a) – Control (untreated cells), (b) – Lipofectamine 3000/pEGFP-N2, (c) – MPI-12(650 μg/ml)/pEGFP-N2, (d) – MPI-16(325 μg/ml)/pEGFP-N2. Red bar confines transfected cells region; percentage value indicates transfection efficacy relative to total cell number. Cells were treated with 4-times diluted complexes and cultured in **α-MEM/10% FBS** for 4 h. Lipofectamin 3000 was applied according to the manufacturer's protocol.

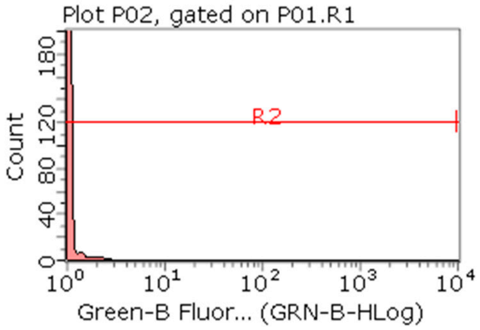

(**a**)

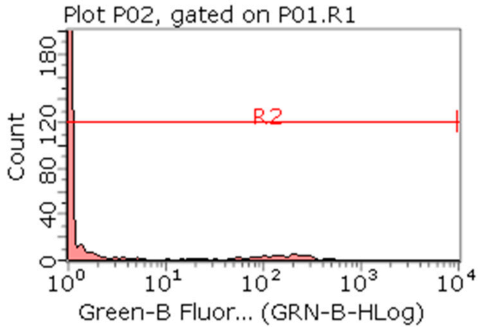

(**b**)

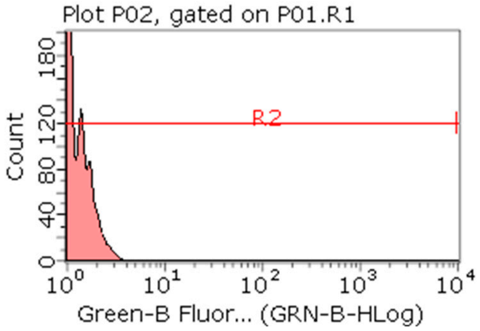

(c)

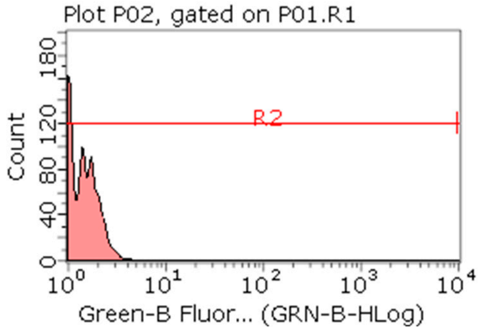

(d)

Figure S7. Distribution of green fluorescence in **M-HeLa cells** cultured in the presence of MPI-n/pEGFP-N2 complexes for 24 h: (a) – Control (untreated cells), (b) – Lipofectamine 3000/pEGFP-N2, (c) – MPI-12(650 μg/ml)/pEGFP-N2, (d) – MPI-16(325 μg/ml)/pEGFP-N2. Red bar confines transfected cells region; percentage value indicates transfection efficacy relative to total cell number. Cells were treated with 4-times diluted complexes and cultured in **α-MEM/10% FBS** for 4 h. Lipofectamine 3000 was applied according to the manufacturer's protocol.

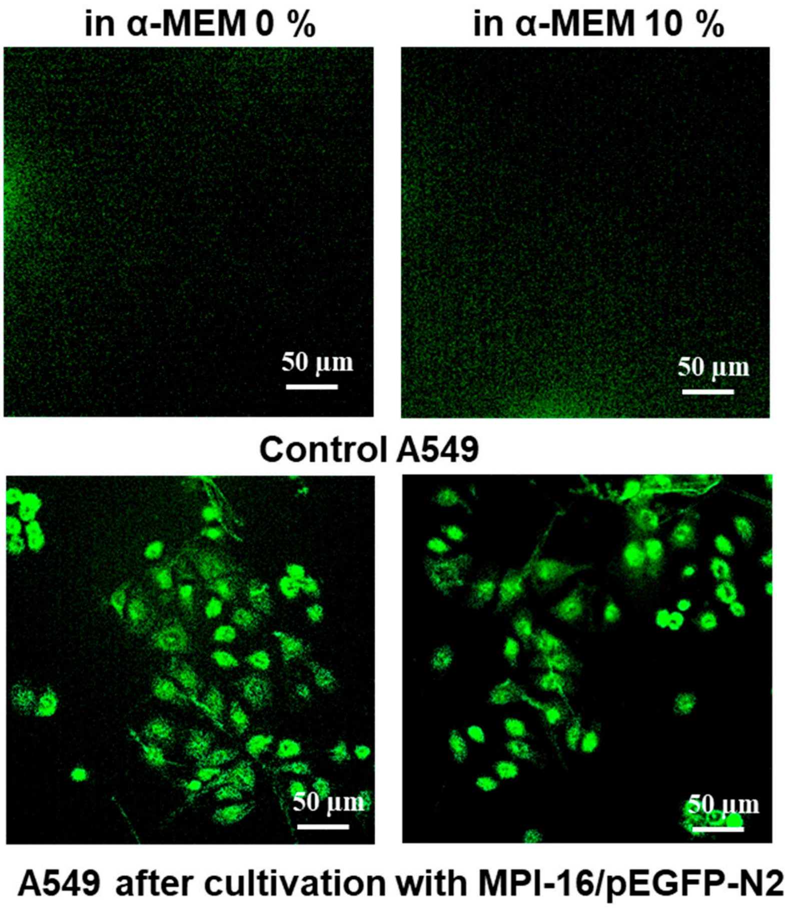

Figure S8. Representative fluorescent microphotographs of A549 cells transfected with MPI-16/pEGFP-N2 complexes under different conditions: 0% α-MEM and 10% α-MEM; cultivation 24 hours.

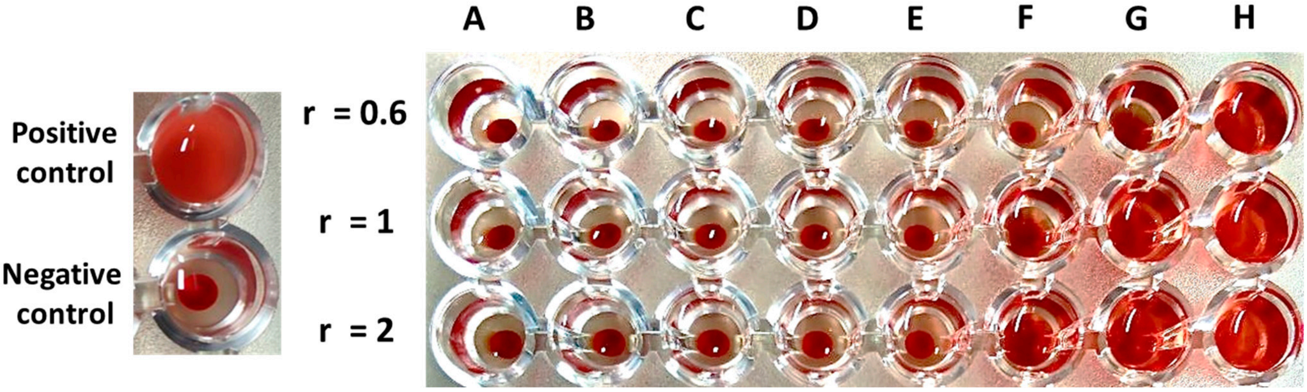

Figure S9. Photograph of microplate wells containing blood samples supplemented with MPI-12/ONu complexes at various *r*.
